# Evolution- and structure-guided redesign of the *Stachel* motif yields soluble and potent modulators of adhesion GPCRs

**DOI:** 10.1016/j.jbc.2026.113323

**Published:** 2026-07-13

**Authors:** Abibe Useini, Yue Feng, Isabell Kaczmarek, Georg Künze, Doreen Thor, Torsten Schöneberg

**Affiliations:** 1 Institute for Drug Discovery, Leipzig University, Leipzig, Germany; 2 Rudolf Schönheimer Institute of Biochemistry, Molecular Biochemistry, Medical Faculty, University of Leipzig, Leipzig, Germany; 3 Department of Pharmacy, Institute of Pharmaceutical Chemistry, University of Marburg, Marburg, Germany; 4 Interdisciplinary Center for Bioinformatics, Leipzig University, Leipzig, Germany; 5 Center for Scalable Data Analytics and Artificial Intelligence, Leipzig University, Leipzig, Germany; 6 School of Medicine, University of Global Health Equity, Kigali, Rwanda

**Keywords:** G protein-coupled receptor, adhesion G protein-coupled receptor, ligand design, structural evolution, GAIN domain, signal transduction

## Abstract

Adhesion GPCRs are activated by an intramolecular agonist known as the *Stachel* sequence. In the intact receptor, this sequence interacts with both the GPCR autoproteolysis-inducing (GAIN) domain and the seven transmembrane domain (7TMD). In contrast, synthetic *Stachel*-derived peptides used in experimental settings only need to bind to the 7TMD to activate the receptor. We therefore hypothesized that natural *Stachel* sequences have evolved under dual structural constraints, interacting with both domains, and that removing the GAIN domain constraint could allow the rational design and optimization of more effective ligands. Using a curated dataset of 13,611 vertebrate adhesion GPCR sequences, we performed large-scale conservation and entropy analyses that revealed strong evolutionary constraint at specific *Stachel* core positions, particularly X^0.47^, X^0.48^, and X^0.50^. Structure-guided *in silico* design with ProteinMPNN, applied separately to GAIN- and 7TMD-bound conformations, generated thousands of *Stachel* variants and demonstrated that single-domain optimization explores sequence space not sampled in natural orthologs. Guided by these analyses, we designed two 11-mer peptides targeting ADGRG2. Both peptides were readily soluble and exhibited substantially increased potency in cAMP assays compared with WT-derived *Stachel* peptides. Structural modeling suggests that backbone constraint at position X^0.45^, polar interactions at X^0.48^, and hydrophobic anchoring at X^0.50^ contribute critically to enhanced 7TMD engagement. Interestingly, analogous design for ADGRF5 did not yield active peptides. Together, our findings support the concept that separating GAIN- and 7TMD-imposed constraints can enable receptor-selective optimization of *Stachel*-derived ligands. This strategy provides a framework for exploring previously inaccessible sequence space, while highlighting receptor-specific trade-offs between potency and specificity.

G protein-coupled receptors (GPCRs) are typically activated by soluble ligands released in an endocrine, paracrine, or autocrine manner. These ligands diffuse and bind to an extracellular pocket formed by the seven transmembrane helices (7TM), inducing a conformational change that enables intracellular interaction of the seven transmembrane helix domain (7TMD) with G proteins. However, some physiological stimuli, such as photons, enzymatic activity, mechanical forces, or high concentrations of proteins that cannot directly access the 7TMD, activate GPCRs indirectly through tethered or intramolecular ligands. Examples include rhodopsin, protease-activated receptors, glycoprotein hormone receptors, and adhesion GPCRs (aGPCRs) (1, 2).

aGPCRs are distinguished by long extracellular N termini, often composed of multiple domains such as immunoglobulin-like, epidermal growth factor–like, leucine-rich, or pentraxin-like domains, which are thought to mediate cell–cell and cell–matrix interactions (3). A defining feature of this GPCR class is the GPCR autoproteolysis-inducing (GAIN) domain, which contains the GPCR proteolytic site (GPS) motif (4). Embedded within the structural framework of the GAIN domain is a short intramolecular agonistic sequence of 10 to 16 amino acids, known as the *Stachel* sequence (5, 6). A large body of structural and experimental evidence suggests that aGPCRs are activated by a *Stachel*-mediated mechanism, in which mechanical forces expose the agonistic sequence, enabling its engagement with the 7TMD (1, 7). Structural studies have further revealed that the *Stachel* sequence can adopt distinct conformations depending on context: within the GAIN domain, it is resolved as a β-strand aligned with the C-terminal β-sheet (4), whereas in several cryo-EM structures of C-terminal fragments (CTFs) in complex with cognate G proteins, it appears as an α-helical intramolecular ligand embedded in the extracellular region of the 7TMD (8, 9, 10).

Early studies highlighted the high evolutionary conservation of *Stachel* motifs across aGPCR paralogs (5), and functional assays demonstrated partial cross-activation by *Stachel*-derived peptides, even across different aGPCR subgroups (11). Currently, synthetic peptide agonists are typically designed as direct copies of native *Stachel* sequences. However, these peptides generally exhibit low affinities for the 7TMD and are often highly hydrophobic (12, 13), resulting in poor solubility and complicating their use in *in vitro* and *in vivo* studies (11). Thus, improved strategies are required to enhance the solubility, potency, and/or specificity of *Stachel*-derived peptide agonists.

In their native context, *Stachel* sequences interact with two parts of aGPCRs, the GAIN domain and the 7TMD. This dual interaction with both domains places strong evolutionary constraints on the amino acid composition of *Stachel* sequences. In contrast, synthetic peptide agonists that are commonly used as experimental probes only need to bind the 7TMD, not the GAIN domain. This difference raises a fundamental question: what structural and evolutionary features allow *Stachel* sequences to interact with both domains? Understanding these constraints could reveal how to modify *Stachel* sequence composition to enhance peptide solubility and receptor specificity.

To address this question, we pursued a stepwise strategy. First, we analyzed whether the GAIN and 7TM domains have coevolved across aGPCRs. Detecting coevolution would indicate that these domains have adapted to each other functionally, imposing shared evolutionary constraints on the *Stachel* sequence. Second, we examined residue-level conservation within *Stachel* motifs to identify whether a common physicochemical pattern exists across all aGPCRs, or whether different receptor subfamilies, and possibly individual receptors, maintain distinct sequence constraints. Next, we used structural modeling with AlphaFold3 (14) and AI-based protein sequence design, such as ProteinMPNN (pMPNN) (15) to predict how individual residues of the *Stachel* sequence contribute to binding the GAIN domain or the 7TMD. These analyses allowed us to pinpoint positions that may represent evolutionary trade-offs, residues conserved because they support interaction with one domain, both, or neither. Finally, we compared residues predicted to be functionally important *in silico* with their degree of evolutionary conservation. This revealed whether certain highly conserved positions, such as Thr/Ser in the GPS motif, contribute directly to binding or serve other structural/functional roles.

Based on the combined evolutionary and structural insights, we designed new activating peptide ligands with optimized solubility and receptor specificity, guided by the natural and *in silico* constraints of *Stachel* sequence evolution.

## Results

### The GAIN and the 7TM domains show strong coevolution

The evolutionary analysis of the 7TMD amino acid sequence of aGPCRs is commonly used to infer phylogenetic relationships, whereas the domain structures of the N termini can vary greatly, particularly among non-vertebrate aGPCR orthologs (16, 17, 18). In fact, the N termini even appear to have been recombined with different extracellular domains in a modular manner. This raises the question of whether the same modular recombination also applies to the GAIN domain of the N terminus, or whether the GAIN domain and the 7TMD have remained genomically linked throughout evolution. If the latter is true, it would suggest that the GAIN domain and the 7TMD are also functionally inseparable.

**Figure 1:**
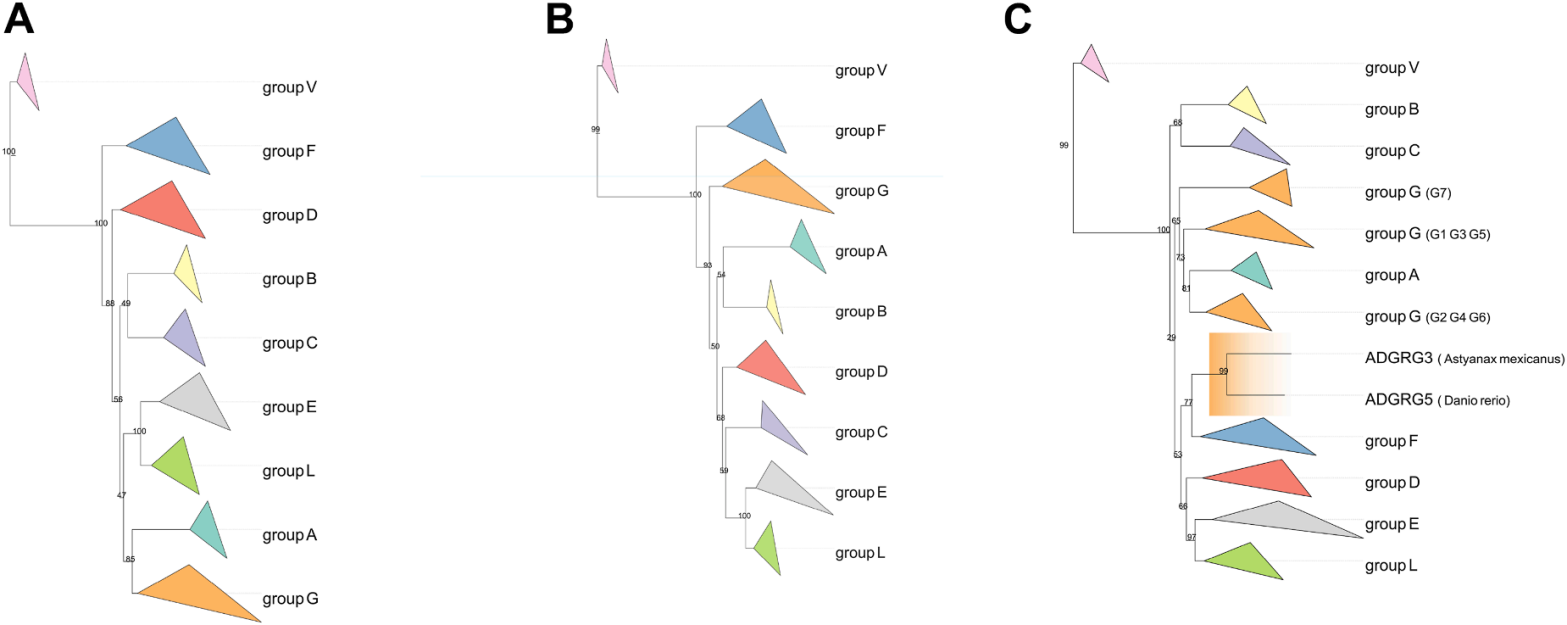
**The coevolution of the GAIN and 7TM domains.** To accurately assign the amino acid sequences of the GAIN plus 7TMD (*A*), the 7TMD alone (*B*), and the GAIN domain alone (*C*) to different aGPCR groups, representatives from all aGPCR families across four vertebrate classes were included in the analysis. Sequence alignments of the indicated domains were used to construct phylogenetic trees with the maximum likelihood method and 10,000 bootstraps, with ADGRV orthologs defined as the outgroup. In the condensed triangle projections of the trees, different groups are color-coded, and bootstrap values >75 are considered significant. The shape of the triangles represents the number of included orthologs and the branch lengths within the groups. The detailed phylogenetic trees are given in Fig. S1. 7TM, seven transmembrane helices; 7TMD, seven transmembrane helix domain; aGPCR, adhesion G protein-coupled receptor; GAIN, G protein-coupled receptor autoproteolysis-inducing domain.

To address this, we generated phylogenetic trees based on (i) the GAIN domain together with the 7TMD, (ii) the 7TMD and, (iii) the GAIN domain alone. As shown in Figure 1, *A*–*C* (and Fig. S1), the GAIN domains (Fig. 1*C*) cluster according to their respective groups, similar to the trees generated using the GAIN plus 7TMD (Fig. 1*A*) or the 7TMD alone (Fig. 1*B*). Observing consistent tree topologies (average RF distance <0.05) across replicates confirmed the stability of our phylogenetic inferences. Although the phylogenetic trees derived from the GAIN domain, 7TMD, and concatenated GAIN–7TMD sequences are not identical at the level of individual branches, they consistently recover the same major aGPCR groupings. Differences in fine-scale topology are expected, as domain-specific analyses contain fewer informative sites and are therefore more sensitive to stochastic variation and lineage-specific rate differences, whereas the concatenated alignment provides greater phylogenetic signal and higher resolution. For example, there is the separation of the entire G group into isolated subclusters, G1/G3/G5, G2/G4/G6, and G7, in the GAIN domain (Fig. 1*C*). This subclustering has been described previously (17, 18). Importantly, the agreement in higher-order clustering across independent datasets supports a shared evolutionary framework linking the GAIN domain and 7TMD rather than arguing against it. Together, these observations are consistent with coordinated evolutionary constraints across both domains within aGPCR groups.

### Determination of *Stachel* sequence conservation

**Figure 2:**
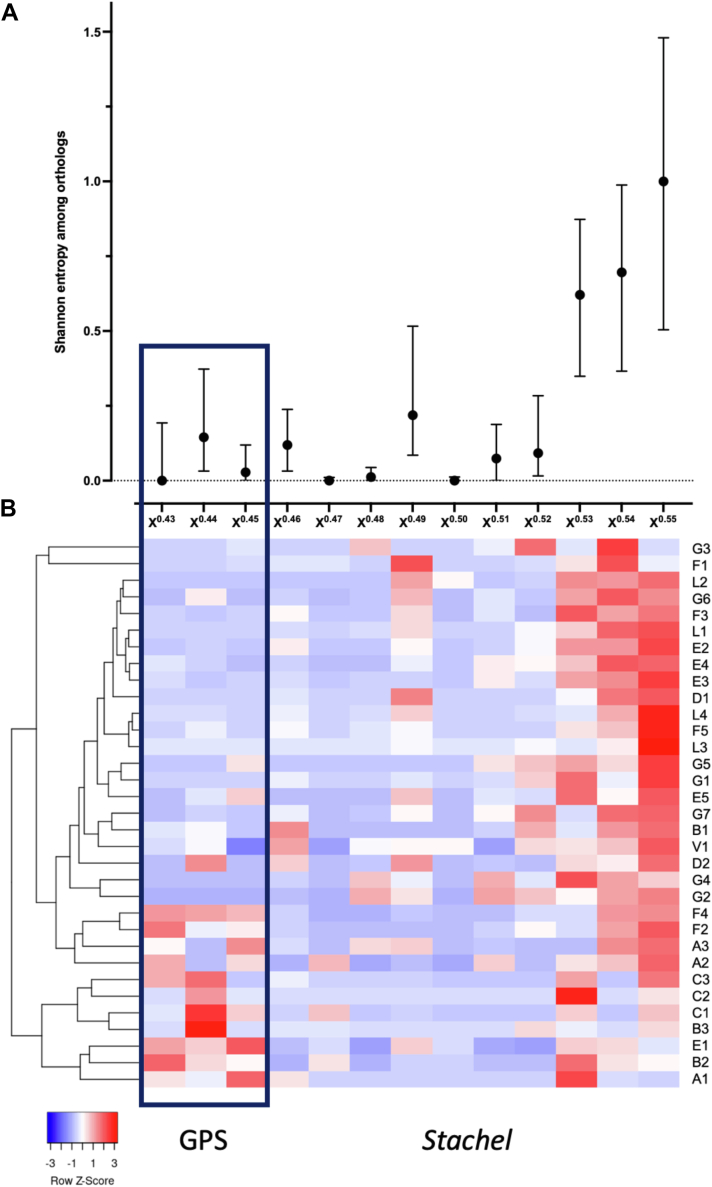
**The conservation of the GPS and *Stachel* sequences in vertebrate *aGPCR*.** Orthologous amino acid sequences of vertebrate adhesion GPCRs (13,611 sequences in total) were retrieved from NCBI and assigned to their corresponding 33 mammalian aGPCR paralogs (*e.g.*, ADGRL1–4, ADGRG1–7, ADGRA1–3, *etc.*). For each paralog, a separate multiple amino acid sequence alignment of vertebrate orthologs was generated. Positional entropy within the GPS and *Stachel* regions was calculated independently for each of the 33 alignments. Residue numbering follows the system described in reference (1), where “X” denotes position relative to the conserved reference framework and X^0.50^ represents the most conserved position across vertebrate aGPCRs. *A*, median positional entropy values across the 33 paralog-specific alignments are shown, with error bars indicating 95% confidence intervals reflecting inter-paralog variability. *B*, the *lower panel* shows positional conservation patterns across aGPCR groups. Cluster analysis (average linkage using Pearson distance) was applied to identify group-specific conservation signatures. High entropy (*red*) indicates low conservation, while low entropy (*blue*) indicates strong conservation. Notably, strong conservation is observed at positions X^0.47^, X^0.48^, and X^0.50^, whereas elevated variability is present within the GPS region (X^0.43^–X^0.45^) and at the C-terminal *Stachel* positions. aGPCR, adhesion G protein-coupled receptor; GPCR, G protein-coupled receptor; GPS, G protein-coupled receptor proteolytic site; NCBI, National Center for Biotechnology Information.

Based on amino acid sequence data as well as functional and structural studies, we and others have recently identified the key residues within the *Stachel* sequence (1, 19). These earlier analyses, however, were based on a limited number of orthologs and paralogs, some of which were not curated for pseudogenes (reflecting relaxed constraint) and showed sampling bias (with a skewed representation of paralogs). In the present study, we used a curated dataset comprising 13,611 vertebrate aGPCR amino acid sequences, excluded obvious pseudogenes, and included in the final analysis only sequences reflecting the relative abundance within the individual ortholog sets of 33 aGPCR members. As a measure of conservation and diversity within the GPS and *Stachel* sequence, we calculated the entropy of each position (Fig. 2*A*).

The GPS includes residues X^0.43^–X^0.45^ and is described in the literature as the HXS/T motif (20). Autoproteolytic cleavage requires a histidine residue together with a serine or threonine residue, with cleavage occurring directly N-terminally to the serine/threonine. As shown in Figure 2*B*, about one third of aGPCRs display higher variability in the GPS motif, indicating a potential lack of autoproteolysis. Because the entropy analysis did not account for the fact that both serine and threonine residues can support autoproteolysis, we manually inspected more than 13,600 sequences and found that 30.6% of all investigated aGPCRs (28.7% corrected for the individual 33 GPCR members, Table S3/Evolutionary constrain) are predicted not to undergo autoproteolysis due to the absence of H^0.43^ and/or S/T^0.45^. Only 17 out of 33 aGPCR members show almost complete conservation of the HXS/T motif across their vertebrate orthologs, indicating that autocleavage is probably not a prerequisite for activation or follows a noncanonical autocleavage mechanism in many aGPCRs.

The *Stachel* sequence has previously been subdivided into a *Stachel* core (interacting mainly with the GAIN and 7TMD) and a *Stachel* linker, a more variable region connecting the *Stachel* core to the first transmembrane helix (1). We analyzed the entire *Stachel* core of 11 amino acid residues of all aGPCR groups and found that the last three positions (X^0.53^–X^0.55^) already exhibit high variability (high entropy), whereas only X^0.47^, X^0.48^, and X^0.50^ show very low entropy, indicating strong conservation (Fig. 2*A*). At the individual group level, there are some aGPCR groups, which are deficient of autocleavage, and show low conservation of the first three positions (residues X^0.43^–X^0.45^). These features and other pattern lead to some clustering of the *Stachel* sequence conservation of aGPCR groups (Fig. 2*B*). Because uneven sampling could influence absolute entropy values, we reperformed the analysis with a subset of aGPCRs equally representing the groups. As shown in Fig. S2, the analysis revealed essentially the same result as the analysis with all investigated vertebrate aGPCR members.

**Figure 3:**
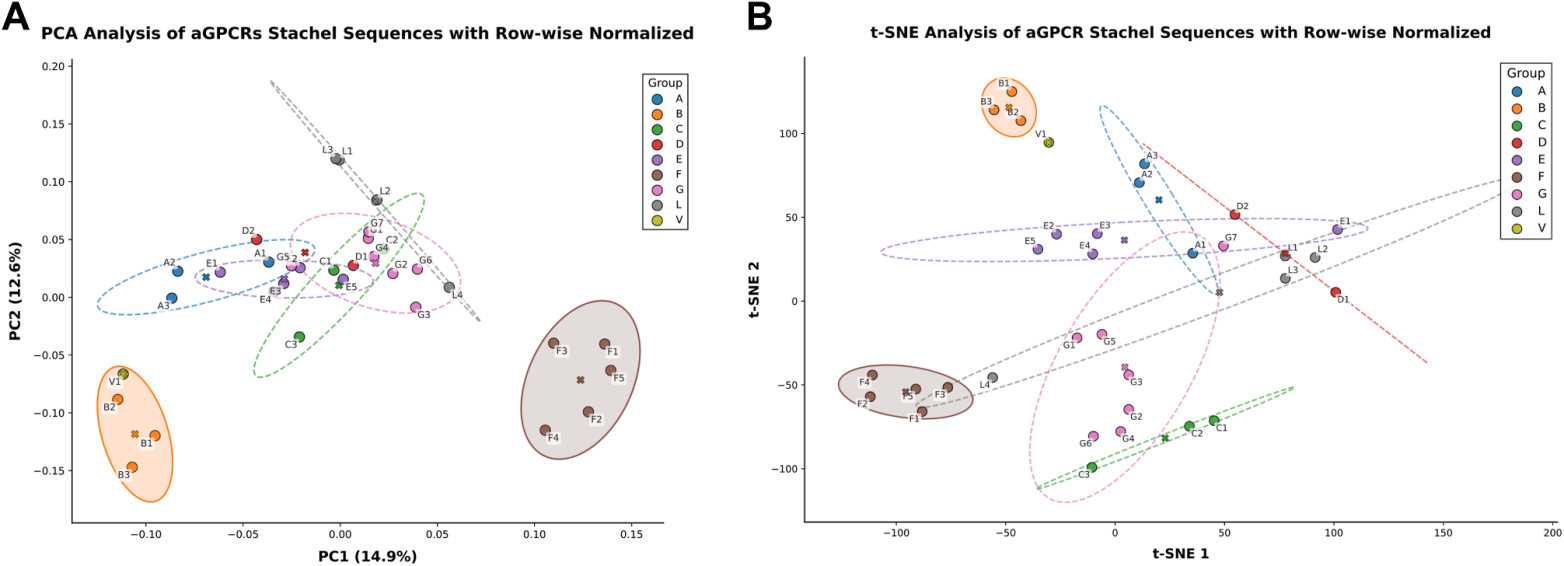
**PCA and t-SNE analysis of the amino acid residue frequency at their positions in the GPS/*Stachel* sequences of vertebrate aGPCR.** To compare amino acid substitution patterns among aGPCR members, the corresponding vertebrate orthologs were aligned, and the amino acid residue frequency was determined at each position in the GPS/*Stachel* sequences. The principal component analysis (PCA) and the t-SNE analysis integrated four parameters: aGPCR member, position within the GPS/*Stachel*, amino acid residue, and residue frequency. *A*, the PCA of these parameters is shown for each aGPCR member. *B*, the t-SNE analysis of these parameters is shown for each aGPCR member. Clusters of paralog groups are marked with *solid circles*, while areas occupied by group members without clear separation from other groups are shown with *dashed circles*. aGPCR, adhesion G protein-coupled receptor; GPS, G protein-coupled receptor proteolytic site; t-SNE, t-distributed stochastic neighbor embedding.

Since entropy is an integrative measure of variability across a dataset but does not specify which residues contribute to the variability, we performed dimensional reduction techniques a principal component analysis (PCA) and a t-distributed stochastic neighbor embedding (t-SNE) analysis integrating four parameters (dimensions): i) aGPCR member, ii) position within the GPS-*Stachel*, iii) different amino acid residues present in orthologs, and iv) the respective frequency of the amino acid residues at each position. With these analyses, we wanted to determine how closely the *Stachel* sequences are related based on these parameters. As shown in Figure 3*A*, the members of most aGPCR groups occupy a compact region. However, members of the B, F, and V groups cluster separately. The t-SNE (Fig. 3*B*) further supports the unique amino acid residue patterns in groups B, F and V.

To identify the amino acid patterns responsible for the separation of certain aGPCR members from the others, we analyzed their GPS/*Stachel* sequences. Specifically, we calculated the average frequency of each amino acid at every sequence position across all aGPCR members. As shown in Figure 2, positions X^0.43^ to X^0.45^ correspond to the GPS with the autocleavable consensus sequence H-X-S/T. Position X^0.46^ is predominantly occupied by hydrophilic amino acid residues (N, T, S, and H; see Table S3/Evolutionary constrain). Positions X^0.47^ to X^0.51^ are highly conserved, mainly hydrophobic (F/Y, A/G, V/I, and L; M/L/A; see Table S3/Evolutionary constrain), and constitute the *Stachel* core sequence of five amino acid residues. Subsequent positions are less conserved and exhibit diverse physicochemical properties.

Although the GPS/*Stachel* sequence pattern of most aGPCRs clusters together (Fig. 3), the amino acid compositions of the B, F, and V groups appear to differ (Table S3/Evolutionary constrain). To depict the position underlaying these differences, we exemplarily plotted the amino acid frequency patterns of ADGRC1 (representing most aGPCR members, Fig. S3*A*) as well as ADGRF4 and ADGRV1 (Fig. S3*B*, right panels), and found that the lack of cleavage-supporting residues, together with substitutions at the highly conserved *Stachel* core (*e.g.*, most aGPCR: Val^0.49^/Leu^0.50^, ADGRV1: Tyr^0.50^, ADGRF4: Ile^0.49^) mainly contributes to their escape from the major cluster (Fig. S3*A*).

### *In silico* design of *Stachel* sequence mutants and their binding abilities to the GAIN domain and the 7TMD

Comparative analysis of thousands of *Stachel* sequences revealed determinants that appear critical for the structure and function of this intramolecular agonistic motif. Since the *Stachel* sequence interacts with both the GAIN and 7TM domains, the contribution of individual residues and their substitutions cannot easily be assigned to specific interactions with one domain or the other. This distinction is of great importance because *Stachel*-derived peptides are typically designed to interact exclusively with, and activate, the 7TMD. To dissect the relevance of individual positions for interactions with either the GAIN or the 7TMD, systematically mutated peptides would need to be tested for binding to both domains separately. In a comprehensive experimental approach, each position would be individually substituted with every possible amino acid (11∗20 = 220 peptides). Considering that combinations of substitutions might also permit or enhance binding, the theoretical number of variants (11^20^ peptides) would be infeasible to synthesize and test experimentally.

Recent advances in structure prediction and protein design tools, such as AlphaFold (21) and pMPNN (15) now allow for the generation and evaluation of hundreds of thousands of ligands *in silico*. We therefore used pMPNN, a new open-source pipeline for protein design, which enables the computational generation and testing of peptide variants from a given sequence with improved solubility and binding properties to a specified target protein structure.

**Figure 4:**
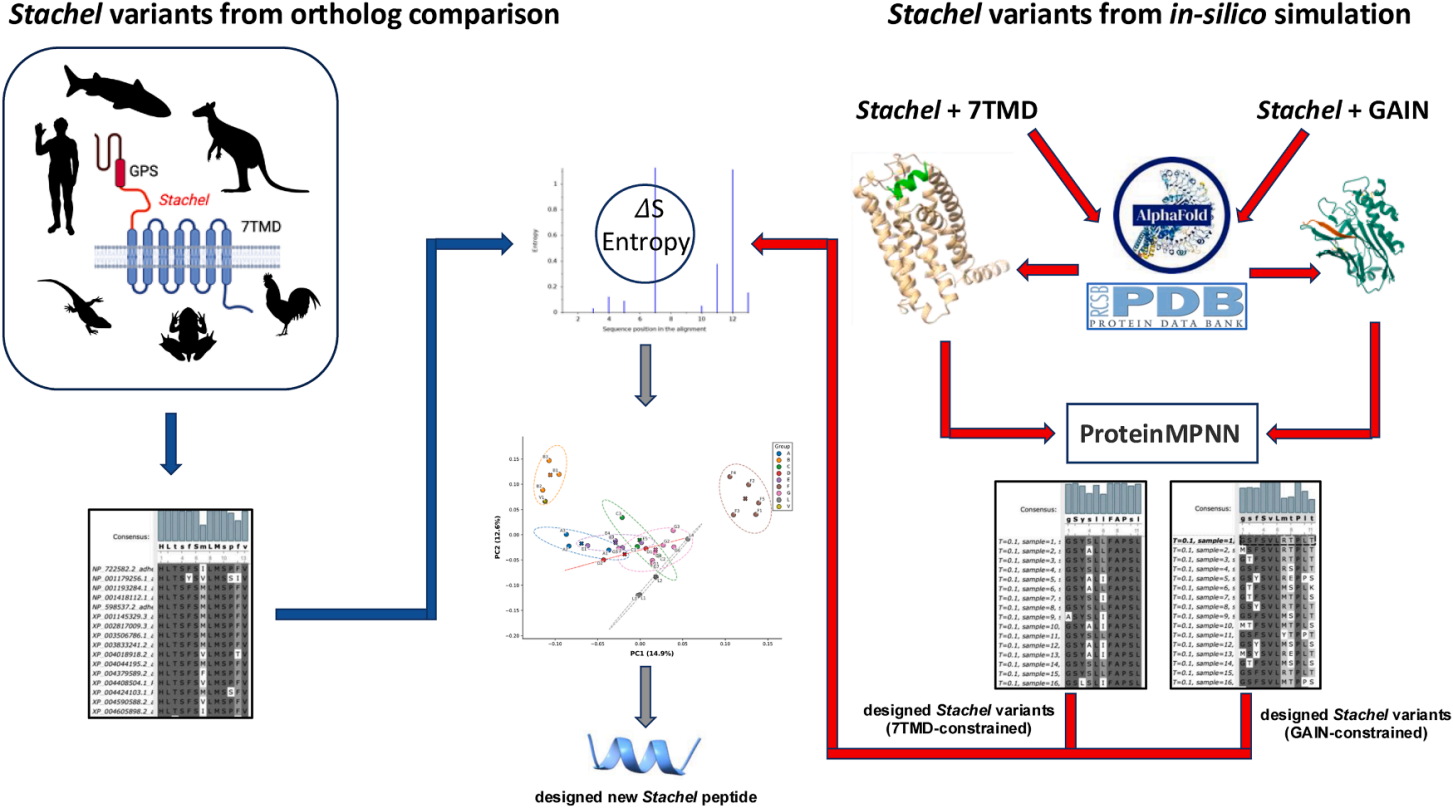
**Workflow for identification and design of *Stachel*-derived peptide agonists.** Orthologous sequences of aGPCRs were aligned and per-position conservation/entropy of the conserved *Stachel* motif was computed. Structural information (experimental structures (from PDB) and AlphaFold models) for the receptor’s GAIN domain and 7TMD were used as inputs to ProteinMPNN to design *Stachel*-sequence variants constrained either to the 7TMD (7TMD-constrained designs) or to the GAIN domain (GAIN-constrained designs). Outputs included per-position entropy profiles extracted from ortholog alignments, novel *Stachel* sequence variants generated by ProteinMPNN under each constraint set, and multivariate summaries (PCA and t-SNE) comparing designed and natural sequences across aGPCRs. Candidate peptides were prioritized based on sequence conservation, ProteinMPNN results, and PCA/t-SNE clustering. 7TMD, seven transmembrane helix domain; aGPCR, adhesion G protein-coupled receptor; GAIN, G protein-coupled receptor autoproteolysis-inducing domain; PCA, principal component analysis.

**Figure 5:**
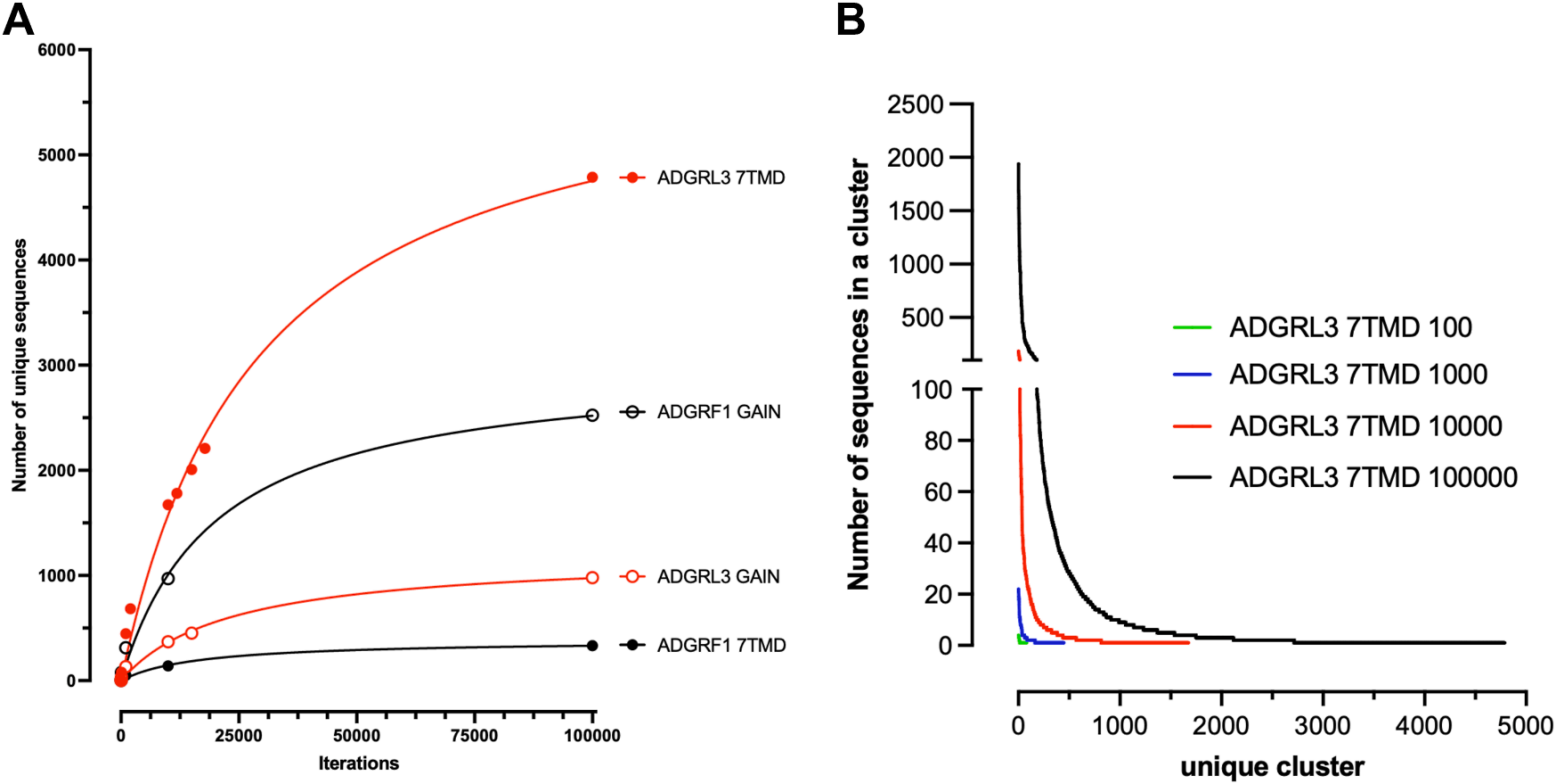
***In silico* generation of *Stachel* sequence variants restricted to the GAIN domain and the 7TMD.***A*, the number of clusters containing unique sequences obtained from pMPNN (15) depending on the number of iterations is plotted. Thus, 11 amino acid residues of the *Stachel* sequence substituted to any other possible proteinogenic amino acid so that the fold of the *Stachel* would structurally fit into binding site of experimentally determined structures of the 7TMD (PDB: 7SF7) and GAIN domain (PDB: 8VTI) of the human ADGRL3 (*red symbols* and *lines*). The same analysis was done for ADRGF1 (*black symbols* and *lines*, 7TMD: PDB 7WXU, GAIN: PDB: 8HC0). *B*, the number of identical sequences within a unique cluster (exemplarily given for the 7TMD analysis of ADGRL3) are plotted depending on the number of iterations (100–100,000). 7TMD, seven transmembrane helix domain; GAIN, G protein-coupled receptor autoproteolysis-inducing domain.

First, we established and validated a workflow to mutate 11 positions of the *Stachel* sequence and to assess binding to experimentally determined structures of the GAIN domain and the active 7TMD (Fig. 4). Although agonistic peptides of 7 to 16 amino acid residues length have been identified from aGPCR (5, 6, 11, 22), we have chosen the first 11 positions of the *Stachel* sequence because this amino acid stretch was always characterized in GAIN structures and it contains the main sequence part interacting with the 7TMD in all available CTF structures (1). In the pMPNN simulations, the receptor’s structure and sequence and the *Stachel* peptide’s backbone structure were kept constant, while the *Stachel* peptide sequence was redesigned. Among human aGPCRs, only two (ADGRF1 and ADGRL3) have experimental structures available for both the GAIN domain (PDB: 8HC0, 8VTI (23, 24)) and 7TMD (PDB: 7WXU, 7SF7 (8, 25)). For our analyses, we used the 7TMD structure bound to the *Stachel* sequence together with the respective GAIN domain of ADGRF1 and ADGRL3 for iterative testing. The number of samplings was successively increased from 10 to 100,000 sequences, and the number of unique sequences binding to the GAIN or the 7TMD was quantified. As shown in Figure 5*A*, increasing the number of iterations led to a rise in unique sequences following a saturation curve. For ADGRF1, interaction of the *Stachel* sequence with the GAIN domain yielded significantly more unique variants reaching a saturation plateau compared to those interacting with the 7TMD. In contrast, for ADGRL3, the interaction of the *Stachel* sequence with the 7TMD generated significantly more unique variants, reaching the saturation threshold, relative to the pMPNN calculation conducted on the GAIN domain.

Many unique amino acid sequences were identified multiple times across iterations. Identical sequences were grouped into clusters, and the number of sequences per cluster was determined. As shown in Figure 5*B* for the ADGRL3 *Stachel*–7TMD interaction, only a few clusters contained many identical sequences, whereas most unique sequences appeared only once. These pilot experiments indicate that ∼10,000 iterations are sufficient to capture the majority of unique binders.

Next, all extracted sequences, both unique and recurring, were aligned to determine which positions tolerated substitutions and which did not. Shannon entropy was again used to quantify positional variability in the *Stachel* sequence of ADGRF1 (Fig. S4, *A* and *B*) and ADGRL3 (Fig. S4, *C* and *D*). It should be noted that Shannon entropy in this context reflects tolerated sequence diversity under design constraints and does not directly quantify binding affinity. For ADGRF1 the 7TMD imposed stronger sequence constraints than the GAIN domain (Fig. S4, *A* and *B*), whereas for ADGRL3 the opposite was observed (Fig. S4, *C* and *D*). In ADGRF1, only position X^0.50^, and in ADGRL3 positions X^0.46^, X^0.47^, and X^0.50^, were constrained by both the GAIN and 7TMD domains. In both cases, the last two positions were less critical for *Stachel* binding to either domain (Table S3/Simulation GAIN/Simulation 7TMD).

### Determination of GAIN- and 7TMD-constrained positions in the *Stachel* of all human aGPCRs

We next applied our *in silico* analysis pipeline to the GAIN and 7TM domains of all human aGPCRs. Experimentally determined human structures were used when available; otherwise, AlphaFold3 models were generated from the corresponding amino acid sequences. As described previously for ADGRF1 and ADGRL3, *in silico* substitution simulations of the *Stachel* sequence in complex with the respective GAIN and 7TMD models were performed for each human aGPCR using 10,000 iterations in pMPNN. Analogous to the analyses of ADGRF1 and ADGRL3, we quantified the number of unique *Stachel* sequence variants and calculated the positional conservation (Shannon entropy) as well as amino acid frequencies across these variants. Eight aGPCR members were excluded from the 7TMD analysis. AlphaFold did not predict models with the *Stachel* sequence bound within the 7TMD for members of the A, B, and V subgroups, and no experimentally determined 7TMD structures are currently available for any group A, B, and V members. ADGRE4 represents a pseudogene in humans and in many other mammalian species.

Across all human receptors, the number of unique *Stachel* sequence variants binding to the target was always below 10,000, ranging from a maximum of 1709 (ADGRC3 7TMD) to a minimum of 30 (ADGRC3 GAIN). In general, the number of unique *Stachel* sequence variants was significantly higher for 7TMDs compared to GAIN domains (mean: 492 *versus* 188; *p* = 0.007, paired *t* test, N = 25), indicating stronger structural constraints imposed by the GAIN domain (Fig. S5*A*). Similarly, sequence conservation (% consensus, Fig. S5*B*) was higher for GAIN-conditioned than for 7TMD-conditioned *Stachel* sequence variants (91% *versus* 86%; *p* = 0.001, paired *t* test).

Interestingly, none of the ∼0.5 million *in silico*-generated sequences was identical to any natural *Stachel* sequence identified among orthologs. These findings are consistent with the idea that natural *Stachel* sequences are shaped by simultaneous constraints imposed by both domains. The resulting natural sequence therefore represents a compromise between these two sets of constraints and differs from sequences predicted when only a single constraint is applied. However, this does not exclude the possibility that additional constraints, such as those related to folding, processing, or biosynthesis, also influence sequence evolution.

As noted previously, approximately 30% of aGPCRs are predicted not to undergo autoproteolysis due to the absence of H^0.43^ and/or S/T^0.45^. It remains unclear whether these receptors follow the canonical activation mechanism involving autoproteolysis, GAIN dissociation, and transition to the 7TMD-bound state. Interestingly, for seven of the twelve aGPCRs lacking canonical GPS motifs (Table S3/noncanonical GPS; groups A, B, and V), AlphaFold did not generate models in which the *Stachel* sequence was embedded in the 7TMD.

**Figure 6:**
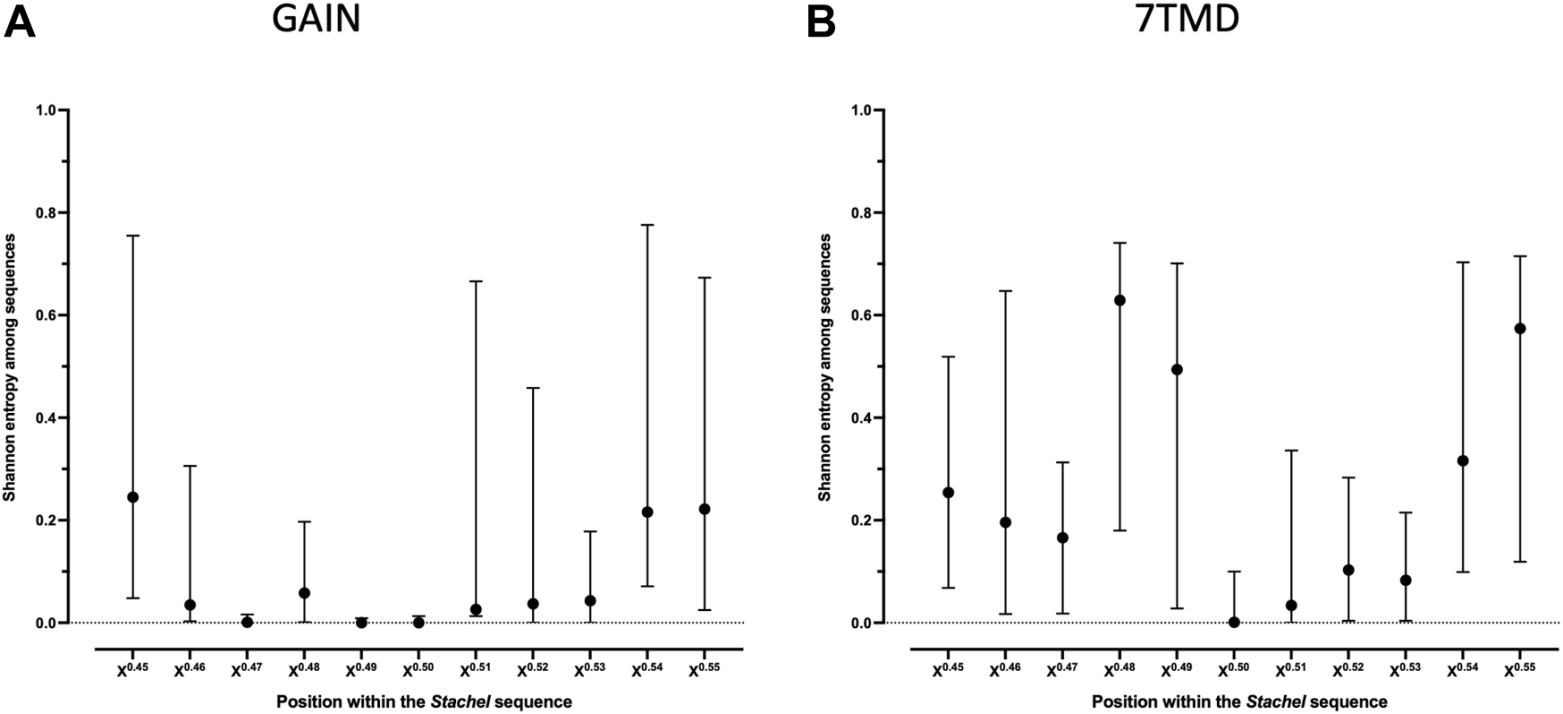
**The entropy of the *Stachel* sequence variants in *in silico* mutated and tested aGPCRs.** Amino acid sequences of the *Stachel* of all 25 analyzed aGPCRs which were found to bind to the GAIN domain (cleaved) (*A*) and the 7TMD (*B*) were aligned (data from 10,000 iterations). From the alignments, the entropy of every position within the *Stachel* sequence variants was calculated. The consensus sequences were different between the 7TMD and the GAIN domain restricted *in silico*-tested *Stachel* sequence is given at the X axes. The median and the 95% confidence interval are shown. 7TMD, seven transmembrane domain; aGPCR, adhesion G protein-coupled receptor; GAIN, G protein-coupled receptor autoproteolysis-inducing domain.

Because our data indicate distinct constraints on *Stachel* binding by the GAIN and 7TMD, we next sought to identify residues contributing most to binding of each domain. As shown in Fig. S5*C* and in more detail in Figure 6, *A* and *B*, the residue-specific constraints differ markedly between the GAIN- and 7TMD-bound *Stachel* sequences. Only position X^0.50^ showed partial overlap. Most other positions displayed a wide range of entropy values, suggesting receptor-specific sequence patterns that may define *Stachel*–7TMD interactions (the detailed entropy values are given in Table S3/Simulation GAIN/Simulation 7TMD). Again, the entropy was significantly lower for GAIN-conditioned than for 7TMD-conditioned *Stachel* sequence variants (0.253 *versus* 0.368; *p* = 0.0007, paired *t* test). This was true also for a subsampled dataset (0.258 *versus* 0.421; *p* = 0.016) containing only eight receptors representing most of the aGPCR groups (except of group A, B, and V; see above) (Fig. S6).

The *Stachel* position X^0.50^ is highly conserved in most aGPCR orthologs and in the simulation. Thus, we ask the question which positions in the GAIN and the 7TMD constrain L^0.50^.

**Figure 7:**
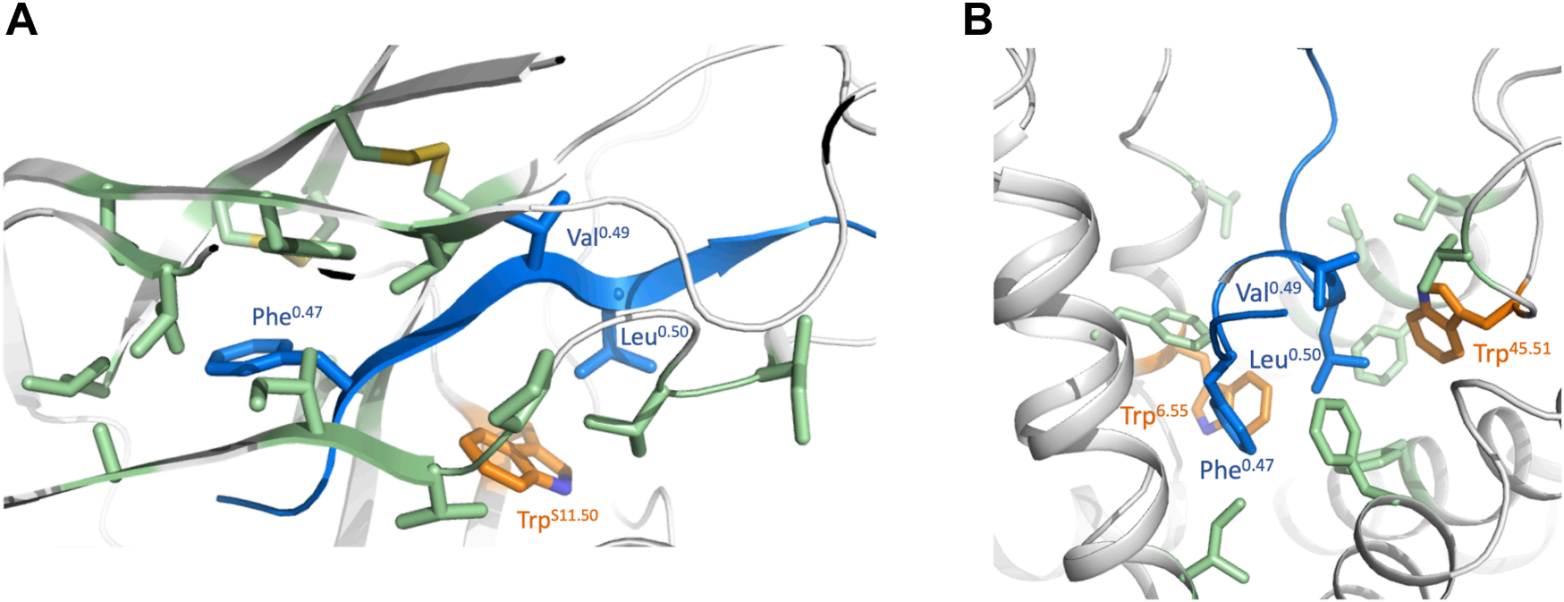
**Structural environment of the GAIN and the 7TMD constraining Leu^0.50^ in the *Stachel* sequence.***A*, the structure of the *Stachel* sequence of human ADGRL3 (*blue*) is shown within the structural environment of GAIN subdomain *B* (PDB 8VTI). The three residues exhibiting the lowest entropy in pMPNN simulations (see Fig. 6*A*) (Phe^0.47^, Val^0.49^, Leu^0.50^) are highlighted. In this cryo-EM structure, the *Stachel* sequence forms a β-strand, in which Phe^0.47^ is embedded between hydrophobic residues (*green*) of adjacent β-sheets within subdomain B. Val^0.49^ points toward a conserved cysteine bridge of this subdomain. Leu^0.50^ interacts with the highly conserved Trp^S11.50^ of β-strand 11. This interaction appears to contribute significantly to binding constraints, as both residues are nearly 100% conserved among vertebrate aGPCRs and adopt this structural arrangement in all available GAIN domain experimental structures. *B*, pMPNN simulations of the *Stachel*–7TMD interaction revealed that only Leu^0.50^ exhibits extremely low entropy (Fig. 6*B*). In the cryo-EM structure of ADGRL3 (PDB 7SF7), Leu^0.50^ interacts with the highly conserved Trp^45.51^ and Trp^6.55^ located in ECL2 and TM6, respectively. Phe^0.47^ forms hydrophobic interactions with residues in TM1 and TM7. 7TMD, seven transmembrane helix domain; aGPCR, adhesion G protein-coupled receptor; ECL2, extracellular loop 2; GAIN, G protein-coupled receptor autoproteolysis-inducing domain; pMPNN, ProteinMPNN.

One would expect that those residues are also highly conserved among all aGPCRs. Detailed inspection of the PDB-derived GAIN domain structures and AlphaFold-predicted GAIN models revealed a Trp residue in ß-strand 11 that is almost 100% conserved among aGPCR ortho- and paralogs (Trp^S11.50^) (Fig. 7*A*). Moreover, V^0.49^ and F^0.47^ are embedded in a hydrophobic environment within subdomain B, although none of the residues constraining V^0.49^ and F^0.47^ is 100% conserved. The highly conserved F^0.47^ is tightly packed within this hydrophobic environment. Analysis of the *Stachel*–7TMD binding mode shows that the lowest-entropy residue L^0.50^ is positioned between the highly conserved W^6.55^ of TM6 and W^45.51^ in extracellular loop 2 (Fig. 7*B*). Both residues, which act as conserved hydrophobic interaction partners of L^0.50^, have been previously identified, and W^6.55^ of TM6 was highlighted because of its toggle switch function in many GPCR (1).

To substantiate the observation of pronounced individuality in *Stachel* sequence patterns, we performed PCA and t-SNE to reduce the dimensional complexity of the dataset (*i.e.*, position within the *Stachel* sequence, possible amino acid substitutions, substitution frequencies, and individual aGPCR identity) (Fig. S7). The PCA and t-SNE projections depict each *Stachel* sequence (*e.g.*, B1 = ADGRB1) in a two-dimensional space, where sequences with similar substitution-frequency patterns are positioned in close proximity. For interpreting the cluster analyses, points forming tight clusters represent sequences with highly similar substitution profiles, whereas those that are distant or located on separate “islands” display distinct substitution patterns.

From a biological perspective, such clustering likely reflects conserved constraints acting on the *Stachel* sequence, including shared structural or functional properties and comparable selective pressures. In contrast, dispersed or intermediate receptors may indicate lineage-specific divergence or mixed substitution profiles. Groups that cluster closely share similar *Stachel* compositions and may therefore interact with the 7TMD and/or GAIN domain in analogous ways (*e.g.*, members of groups F and L at the 7TMD; Fig. S7, *A* and *C*). However, the *Stachel*–GAIN domain simulation analysis clearly separated groups F and L (Fig. S7, *B* and *D*), indicating that different constraints act on the 7TMD and GAIN domains. Groups that partially overlap or occupy intermediate positions between clusters may share limited similarities (*e.g.*, conservation at a few key residue positions) and could represent transitional evolutionary states.

Isolated outliers (*e.g.*, specific members of groups C and G) are particularly noteworthy, as they may possess unique *Stachel* sequences that enable maximal functional divergence and facilitate the emergence of novel *Stachel* peptide activities. Notably, the GAIN domain–based sequence iteration yielded a more stringent clustering pattern than that derived from the 7TMD (Fig. S7). In line with the objective of designing aGPCR-specific agonistic *Stachel* peptides, the sequence patterns generated for each aGPCR appear sufficiently distinct to support the rational design of receptor-selective agonists.

In sum, all data indicate that the GAIN domain restricts the *Stachel* sequence more than the 7TMD. Only the 7TMD of most members of the F group and of ADGRG3 and ADGRG7 restrict the *in silico Stachel* variability more than the GAIN domain (Table S3/Simulation GAIN/Simulation 7TMD, unique cluster comparison). The most entropically conserved residue *in vivo* and *in silico* is X^0.50^. Dimension reduction analyses showed that only a few aGPCRs have highly similar entropy patterns of the *Stachel* sequences but most are distinct from each other enabling the design of receptor specific peptide agonists.

### *In silico* design of soluble *Stachel* sequence-derived peptides

**Figure 8:**
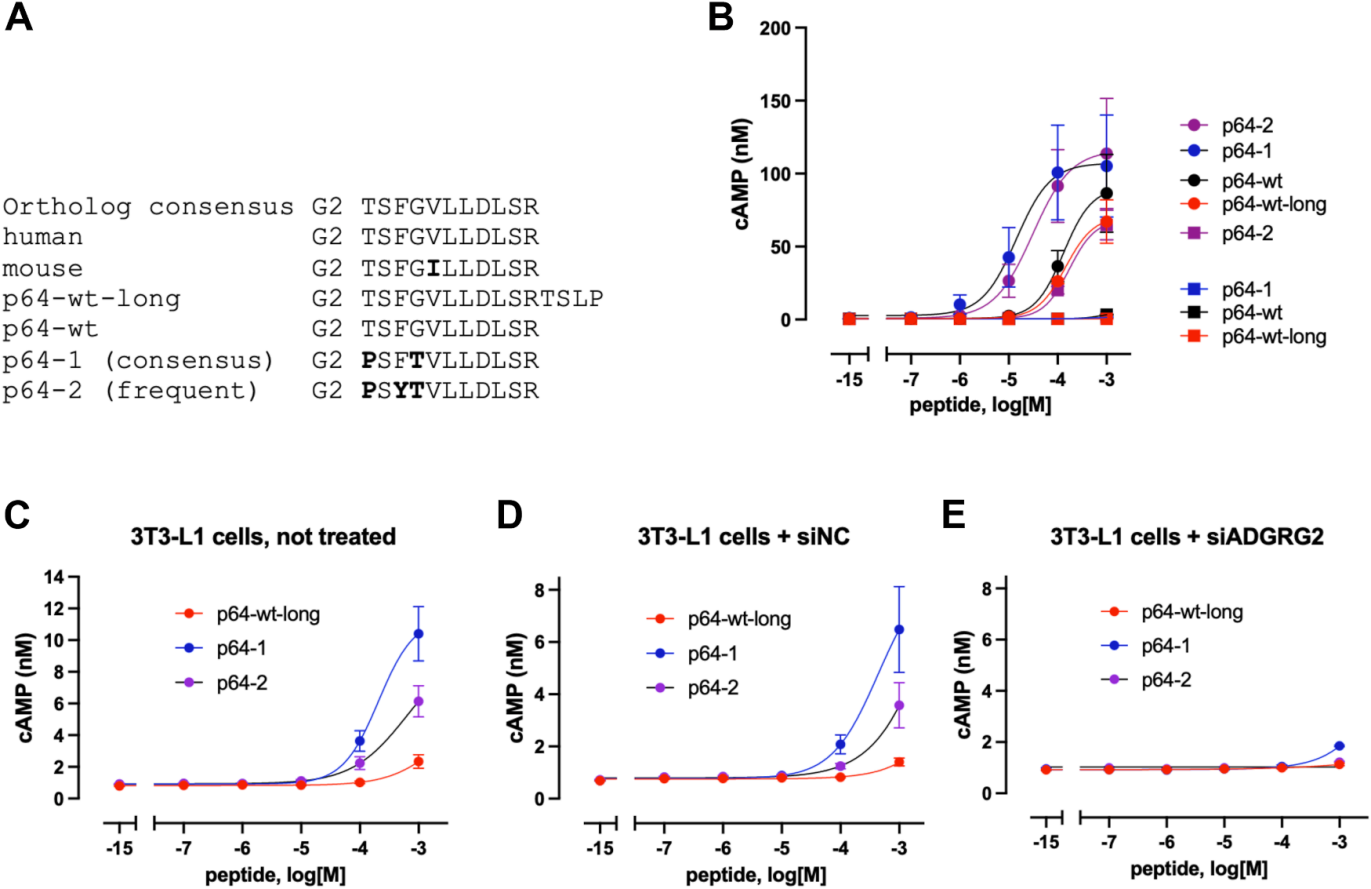
**Functional analysis of ADGRG2-*Stachel* sequence variants generated by simulation.***A*, alignment of 446 vertebrate orthologs revealed an ortholog consensus sequence identical to the human sequence. Mouse ADGRG2 differs only at position X^0.49^ (V to I), where 81.8% of the investigated orthologs contain valine and 18.2% contain isoleucine. Previous work identified *Stachel* sequence-derived peptides as agonists at the mouse ADGRG2 (22). The human ADGRG2 7TMD structure was predicted with Alphafold3, *Stachel* sequence simulations using pMPNN were performed, and peptides p64-1 and p64-2 were synthesized based on the consensus sequence and a highly frequent designed sequence. *B*, HEK293T cells were transiently transfected with mouse ADGRG2, and the indicated peptides were tested in cAMP assays (*circles*). For control purposes, all peptides were also tested in empty-vector (pcDps)–transfected cells (*squares*). Because of significant background activity of the ADGRG2-derived peptides, we performed cAMP assays in the 3T3-L1 preadipocyte cell line, which endogenously expresses ADGRG2 (40). Untreated cells (*C*) and 3T3-L1 preadipocytes transfected with a nonspecific control siRNA (*D*) showed response curves for p64-1, p64-2, and p64-wt that were essentially similar to those observed in HEK293 cells (*B*) and COS-7 cells (Fig. S8). Knockdown of endogenous ADGRG2 using siRNA significantly diminished the activity of the agonistic peptides (*E*). cAMP levels as a function of peptide concentration are shown. Data are presented as mean ± S.E.M. from three independent experiments performed with technical triplicates. 7TMD, seven transmembrane helix domain.

To test whether our approach of separating constraint information may result in improved solubility and/or potency of *Stachel* sequence–derived peptides, we used data from 10,000 iterations of *Stachel*/7TMD simulations of selected aGPCRs to improve agonistic peptides for experimental testing. We applied two rationales for peptide selection. First, we used the consensus sequence derived from all iterations, and second, we selected the most frequently designed peptide sequence. As a representative example, we chose ADGRG2 (GPR64) to test an ortholog- and pMPNN-based prediction of 7TMD-selective agonistic peptides for improved solubility to the 7TMD. This aGPCR is well-characterized in respect to its signal transduction, *Stachel*-peptide activation and physiological relevance (11, 22, 26). Accordingly, the 11-mer *Stachel* consensus peptide (the 11-mer p64-1 peptide; 1385 times found in CTF simulation) and one of the most frequent sequences (the 11-mer p64-2 peptide) from the pMPNN ADGRG2-CTF simulation (1385- and 931-times found) were synthesized (Fig. 8*A*) and tested in concentration–response cAMP assays using ADGRG2-transfected HEK293T cells. For control purposes, empty-vector–transfected cells were included, and two *Stachel* sequence–derived peptides with the WT sequence (p64-wt, 11-mer; and p64-wt-long 15-mer (22)) were also tested.

As expected, the WT *Stachel* sequence–derived peptides (p64-wt-long and p64-wt) showed no cAMP induction in empty-vector–transfected cells and elicited a robust cAMP response at a concentration of 1 mM (Fig. 8*B*). The p64-1 and p64-2 peptides were, like the WT-derived peptides, readily soluble in 1% dimethyl sulfoxide/Dulbecco's modified Eagle's medium (DMEM), respectively, and displayed approximately sevenfold higher potencies (*p* < 0.005) together with higher efficacies (Fig. 8*B*). Because WT peptides (p64-wt and p64-wt-long) did not reach saturation under the tested conditions (more than 1 mM peptide is hampered by solubility), the true potency difference may be larger than indicated by fitted EC_50_ values; however, this cannot be quantified precisely from the current data. This increase in potency was achieved with only two and three amino-acid substitutions in p64-1 and p64-2, respectively (Fig. 8*A*). However, p64-2 showed appreciable cAMP formation in empty-vector–transfected cells, indicating an unidentified cross-reactivity with endogenous targets. Therefore, we reperformed the assays in COS-7 cells and obtained essentially the same results but low background signals in empty vector-transfected cells (Fig. S8). Because residual background activity and potential off-target effects were still observed in COS-7 cells, we tried to validate the pharmacological properties of the ADGRG2-derived peptides using two independent experimental strategies. First, we generated ADGRG2 BRET reporter constructs to directly measure Gs recruitment as a readout of receptor activation. However, as reported for several other aGPCRs, C-terminal fusion of either mVenus or NLuc to ADGRG2 markedly reduced cell surface expression (<3% of WT levels; Fig. S9*A*). Consequently, only weak BRET signals were detected, with WT p64 producing a small response at 1 mM and no significant response at 10 μM (Fig. S9*B*). Testing the reverse BRET configuration and optimizing receptor protein transfection ratios did not improve signal strength. Thus, despite extensive optimization, BRET signals remained too weak to generate meaningful concentration–response curves. Second, we performed functional cAMP assays in 3T3-L1 preadipocytes, which endogenously express ADGRG2 (27). As shown in Figure 8, *C*–*E*, and consistent with results obtained in COS-7 and HEK293 cells, concentration–response curves for p64-1 and p64-2 were significantly shifted toward lower EC_50_ values relative to p64-wt. Importantly, siRNA-mediated knockdown of ADGRG2 in this cell line abolished the responses to all tested peptides, demonstrating that the observed signaling is specifically mediated by ADGRG2 and not attributable to off-target activation of related receptors or paralogs.

We also pursued additional improvements in peptide solubility and affinity by analyzing AlphaFold-modeled ADGRG2-peptide complex structures from more than one hundred uniquely designed *Stachel* peptide sequences, and selecting five peptides with favorable *in silico* scores for experimental testing. However, although solubility was improved, the most promising candidate peptides did not exhibit increased affinity or potency compared with p64-wt (see Supplemental Information and Fig. S10).

To test whether this pMPNN-based prediction approach for 7TMD-selective agonistic peptides is applicable to other aGPCRs, we applied it to ADGRF5 (GPR116), which belongs to a different aGPCR subgroup and couples to Gq proteins. As before, pMPNN analysis of the CTF was performed using 10,000 iterations, and the consensus sequence (p116-1, identified 3353 times in the CTF simulation) was selected to synthesize an 11-mer peptide (Fig. S11*A*). Functional testing of this peptide (p116-1) in ADGRF5-transfected cells revealed no effect on inositol-1-phosphate (IP1) levels upon incubation with concentrations of up to 1 mM, whereas the WT peptide (p116-wt) induced a robust increase in IP1 (Fig. S11*B*). Because p116-1 contained substantial modifications of the core *Stachel* sequence (positions X^0.51^ and X^0.52^; see Fig. 6*A*), and based on our experience with p64 peptides, we reintroduced the amino acid residues present in the WT *Stachel* sequence (M^0.51^ and S^0.52^), yielding peptide p116-2. However, this peptide also showed no agonistic activity (Fig. S11*B*). To assess whether p116-1 and p116-2 still bind to the receptor, p116-wt was co-incubated with either p116-1 or p116-2. As shown in Figure S11*B*, both peptides did not reduce p116-wt–induced IP1 formation and, therefore, possess no competitive antagonistic properties.

## Discussion

Our integrated approach, combining broad evolutionary surveys, structural modeling and large-scale *in silico* sequence design, provides a practical route to reengineer *Stachel*-derived peptides with markedly improved pharmacological properties. The evolutionary analyses showed that natural *Stachel* sequences are shaped by competing constraints: the GAIN domain enforces stringent residue choices at some positions while the 7TMD tolerates greater sequence diversity (or *vice versa*) depending on the receptor (Figs. 6, S5 and S6). Because naturally encoded *Stachel* motifs must satisfy both interfaces, they represent evolutionary compromises. By contrast, pMPNN designs, which are constrained solely by the 7TMD structure, explore sequence solutions that relieve the GAIN-binding constraint and thereby allow substitutions that improve solubility and/or 7TMD engagement. This rationale is supported by the fact that none of the ∼0.5 million *in silico* sequences matched any natural orthologous *Stachel* sequence, underlining that single-domain conditioning moves sequences outside the naturally sampled sequence space.

The ADGRG2 functional data provide a concrete proof of principle for this strategy. Both the pMPNN consensus (p64-1) and the frequent pMPNN design (p64-2) were soluble under assay conditions and elicited substantially stronger and more potent cAMP responses in ADGRG2-transfected cells than the WT-derived peptides p64-wt and p64-wt-long (Fig. 8). The potency shift can be interpreted conservatively: measured EC_50_ values imply roughly a seven-fold potency increase when comparing fitted curves; because the WT peptides did not reach saturation at 1 mM in our assay, potency gains may be as >7-fold when extrapolating maximal efficacies, an important practical advantage when working with peptides that otherwise require millimolar concentrations. These gains were achieved with only two (p64-1) and three (p64-2) amino-acid substitutions relative to the WT, demonstrating that modest, targeted changes guided by structure and design tools can have outsized effects on activity and solubility.

**Figure 9:**
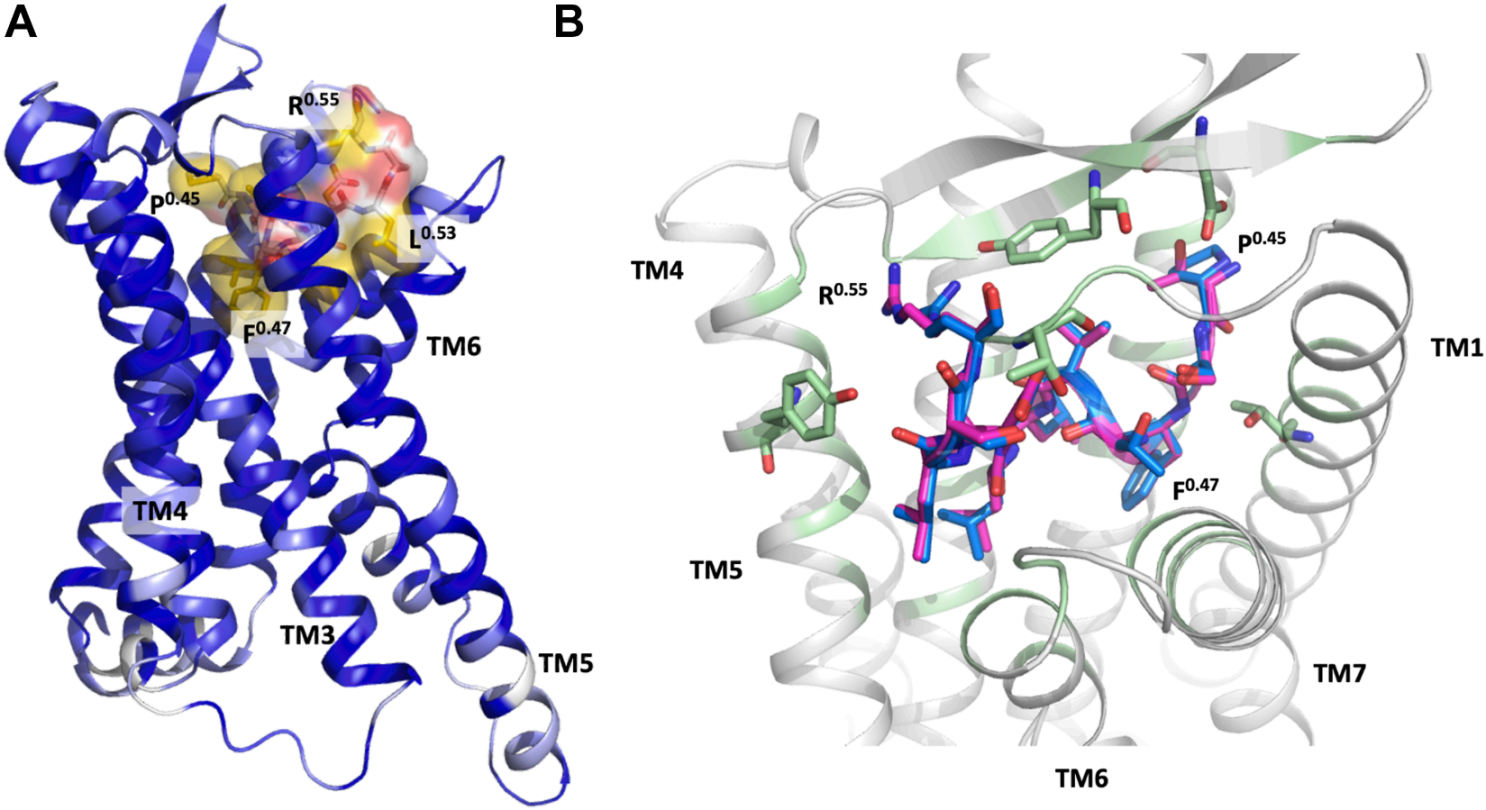
**Structural environment of the agonistic peptide p64-1 *in ADGRG2*.***A*, structural model of ADGRG2 bound to the agonistic peptide p64-1. The hydrophobicity of the 7TMD is shown from *white* (less hydrophobic) to *blue* (more hydrophobic). For p64-1, the molecular surface is colored by physicochemical properties, with hydrophobic regions in *yellow*. In addition, oxygen and nitrogen atoms are colored in *red* and *blue*, respectively. *B*, superposition of the native *Stachel* sequence (*magenta backbone*) and p64-1 (*blue backbone*), respectively, together with the surrounding binding pocket residues of the ADGRG2 7TMD. 7TMD, seven transmembrane helix domain.

Structural analysis and the *in silico* designed sequence spaces converge on three *Stachel* positions as primary determinants of 7TMD engagement for ADGRG2. Introducing a proline at the very N terminus of the *Stachel* (P^0.45^) likely imposes a local conformational constraint, favoring a defined backbone turn or helix capping, that optimizes the presentation of downstream residues to the 7TMD binding pocket. Threonine at position T^0.48^ can form a stabilizing hydrogen bond network with polar side chains or backbone carbonyls in the extracellular vestibule, enhancing specificity without greatly increasing hydrophobicity. Leucine at L^0.50^ provides essential hydrophobic packing into a conserved 7TMD pocket formed by residues such as W^6.55^; this packing is expected to be a major contributor to binding affinity and is consistent with the strong conservation of X^0.50^ across orthologs. Together, these three features, backbone rigidification (P^0.45^), polar side-chain engagement (T^0.48^), and hydrophobic anchoring (F/Y^0.49^, L^0.50^), explain why the designed p64-1/-2 sequences show enhanced potency against the isolated 7TMD relative to WT peptides that also must accommodate GAIN interactions. We propose this mechanistic model in the text and illustrate it in the accompanying figure showing the *Stachel* N terminus docked into the ADGRG2 7TMD pocket (Fig. 9).

The observation that p64-2 produced appreciable cAMP signals in empty-vector–transfected HEK293T cells indicates potential off-target activation of endogenous GPCR(s) or other cAMP-modulating proteins. The reduction of this signal in COS-7 cells suggests cell type–specific expression of the relevant off-target(s). To identify potential targets of p64-2, we evaluated publicly availably RNA-Seq datasets from HEK293T and COS-7 cells employed in our assays (GSE285962, GSE121304) and compared endogenous aGPCR expression profiles as potential off-target candidates (Fig. S12). ADGRG2/GPR64 is expressed at low levels in both cell lines but is slightly more expressed in COS-7 cells than in HEK293T cells. Although this difference might contribute to variation between the two cell lines, it does not explain why p64-1 fails to produce a comparable background signal if endogenous ADGRG2 receptors were responsible for the effect. Thus, if an aGPCR mediates the off-target response, it would likely be a receptor that is higher expressed in HEK293T than in COS-7 cells and is selectively activated by p64-2 but not by p64-1. This is observed for members of the ADGRL group, for which both increases (Gs coupling) and decreases (Gi coupling) in cAMP signaling have been reported (13, 28, 29). However, we did not observe any effect on cAMP levels upon incubation of ADGRL1 or ADGRL3 with p64-2 (data not shown). Based on prior reports of cross-activation by *Stachel*-derived peptides (11), members of the ADGRG group would represent plausible candidates. However, none of these receptors is higher expressed in HEK293T cells than in COS-7 cells. For other aGPCRs displaying higher expression in HEK293T cells (groups A, B, C, and V; Fig. S12), modulation of cAMP signaling has not been reported to date. Finally, we verified the potency and specificity of p64-1 and p64-2 using 3T3-L1 cells, which endogenously express ADGRG2. In this cell system, we observed a clear ADGRG2-dependent shift in potency and specificity (Fig. 8, *C*–*E*).

Practically, this finding underlines an important trade-off in single-domain optimization: relieving GAIN-binding constraints may permit sequence changes that enhance 7TMD potency but also expose physicochemical motifs capable of promiscuous interactions. For future development, p64-1 appears preferable because it combined increased potency with less evidence of off-target activation in our hands; nonetheless, systematic profiling across a panel of GPCRs and broader cell types will be required to define selectivity and safety margins.

Our results suggest two complementary strategies for engineering *Stachel*-derived agonists. The first is to design peptides explicitly constrained to the 7TMD, accepting loss of GAIN compatibility to gain solubility and potency for exogenous applications (drug probes or therapeutics). The second is to seek hybrid solutions that retain minimal GAIN contacts required to avoid off-target recognition while optimizing key 7TMD-engaging positions (for example, preserving hydrophobic anchors like L^0.50^ but introducing polar residues elsewhere to improve solubility). More generally, the work highlights how evolutionary analyses can reveal which residues are likely to tolerate substitution and which will disrupt essential intramolecular contacts; this information helps prioritize substitutions with the highest probability of improving biophysical properties without destroying receptor specificity.

Several caveats temper our conclusions. First, the pMPNN designs were generated using static receptor conformations (cryo-EM or AlphaFold models) and thus do not fully capture conformational dynamics that may contribute to binding. Second, the solubility and potency improvements observed *in vitro* will not necessarily translate to *in vivo* stability, bioavailability, or pharmacokinetics for therapeutic applications. Third, together with the cross-receptor *in silico* survey, the ADGRG2 data provides a positive example that decoupling GAIN- and 7TMD-specific constraints can yield potent agonists. However, it is not a proof-of-principle pipeline for other aGPCR. Our testing currently excludes several subgroups (A, B, and V) from 7TMD-conditioned modeling due to a lack of confident embedded-peptide models; broader cross-subgroup testing will be required to assess generality. Moreover, while modeling identifies candidate interactions (P^0.45^, T^0.48^, F/Y^0.49^, and L^0.50^) that plausibly drive 7TMD binding, direct structural validation (co-cryo-EM or high-resolution NMR of peptide–receptor complexes) is required to confirm atomic contacts. Finally, the functional failure of designing *Stachel*-derived peptides for ADGRF5 suggests that for certain receptors, the *Stachel*–7TMD interaction may require conformational dynamics or GAIN-contextual interactions that are not captured in static structural models.

Our comparison of ADGRG2 and ADGRF5 underscores both the potential and current limitations of structure-guided *Stachel*-peptide design. Whereas peptide optimization was successful for ADGRG2, no active ligands were obtained for ADGRF5, suggesting that the availability of accurate structural information, particularly experimental 7TMD structures, is an important determinant of design success. The functional failure of designing *Stachel*-derived peptides for ADGRF5 suggests that for certain receptors, the *Stachel*–7TMD interaction may require conformational dynamics or GAIN-contextual interactions that are not captured in static structural models. These observations further indicate that receptor-specific features contribute to the outcome and may limit straightforward transferability of design strategies across aGPCR subtypes. More broadly, our results support a framework in which evolutionary conservation identifies structurally constrained residues, while computational design highlights positions amenable to modification. Incorporation of polarity-enhancing residues at variable sites may improve solubility and receptor engagement, although iterative experimental validation remains necessary to achieve optimal activity.

In summary, separating evolutionary constraints imposed by the GAIN and the 7TMD reveals sequence space that is not sampled in nature but is highly useful for designing soluble, potent *Stachel*-derived agonists. The ADGRG2 pMPNN-designed peptides exemplify this principle: with only a few substitutions guided by structural and evolutionary insight (notably at P^0.45^, T^0.48^, F/Y^0.49^, and L^0.50^), we achieved gains in potency and solubility while uncovering important considerations about specificity and off-target risk. This framework can enable rational design of soluble, potent *Stachel*-derived ligands in selected receptors, as shown here for ADGRG2. At present, this represents a receptor-specific proof-of-concept rather than a universally applicable design pipeline. Future work integrating ensemble modeling, explicit membrane representations, and experimental structural validation will be required to extend this strategy across additional aGPCR subgroups.

## Experimental procedures

### Retrieval of aGPCR sequences from databases

All vertebrate aGPCR amino acid sequences used in this study were retrieved from NCBI/GenBank. Highly fragmented sequences or sequences with low quality were excluded from the analyses. In total, 13,611 vertebrate aGPCR amino acid sequences were retrieved from these databases and are listed in Table S1.

### Alignments and phylogenetic analyses

Protein sequences were aligned using the UGENE (v52) analysis tool (30), employing the MUSCLE algorithm (31) with default settings. Alignments for phylogenetic trees were generated using MAFFT (32) under the L-INS-i algorithm with the BLOSUM62 substitution matrix and iterative refinement (16 iterations). All alignments were manually inspected and curated. Evolutionary relationships of the 7TMD, GAIN domain, and concatenated GAIN-7TMD of aGPCRs were inferred using maximum likelihood and Bayesian inference. First, maximum likelihood analysis was performed using IQ-TREE 2 (33). The optimal substitution model (Q.pfam+I+R7 for gain-7TM; WAG+F+R7 for gain domain; Q.plant+I+R7 for 7TM domain) was selected *via* ModelFinder (34) under the Bayesian Information Criterion. Branch support was assessed with ultrafast bootstrap approximation (UFBoot; 10,000 replicates). ADGRV orthologs were designated as the outgroup, and final trees were visualized using TVBOT (35). Second, Bayesian inference was conducted using MrBayes3.2 (36) with a mixed amino acid substitution model incorporating invariant sites and gamma-distributed rate variation. The analysis comprised two independent runs of four Markov chains each for 1,000,000 generations (sampling every 1000 generations). After discarding the first 25% of samples as burn-in, convergence was confirmed by average standard deviation of split frequencies (<0.01). A majority-rule consensus tree was generated from postburn-in samples, again using ADGRV orthologs as the outgroup. To validate topological robustness, we performed triplicate analyses with randomized input orders, observing consistent tree topologies (average RF distance <0.05) across replicates, confirming the stability of our phylogenetic inferences.

### Determination of the conservation and entropy

The conservation of each position in the amino acid alignments was described *i*) by the frequency of the most abundant amino acid residue and *ii*) by determining the entropy of a given position from the alignment. Shannon entropy is a simple quantitative measure of uncertainty in a data set (https://www.hiv.lanl.gov/content/sequence/ENTROPY/entropy_one.html) (37). Thus, a total of 33 mammalian aGPCR paralogs (*e.g.*, ADGRL1–4, ADGRG1–7, and ADGRA1–3) were used as reference groups for the analysis. For each paralog, all available vertebrate orthologous protein sequences were retrieved from the NCBI database, resulting in a combined dataset of 13,611 amino acid sequences. Orthologs were assigned to their respective mammalian paralog, and separate multiple sequence alignments were generated for each of the 33 paralog-specific groups. Positional amino acid entropy was then calculated independently for the GPS and *Stachel* regions within each alignment. To obtain a robust estimate of conservation across the aGPCR family, median entropy values for each position were computed across the 33 paralog-specific alignments, and 95% confidence intervals were derived from the distribution of entropy values across paralogs. This approach allowed assessment of both position-specific conservation and variability between aGPCR groups.

### Simulation of *Stachel* sequence and domain interaction

The 3D structures of the aGPCR domains were obtained either from the PDB or predicted from their amino acid sequences using AlphaFold3 (14). The structures used as input models are listed in Table S2. All structures were prepared for subsequent calculations by removing domains and regions other than the 7TMD/GAIN and *Stachel* interface.

For each receptor analyzed in this study, two design strategies were applied to the *Stachel* sequence. Either the 7TMD or the GAIN domain served as the target structure, and the *Stachel* sequence was redesigned while bound to the 7TMD (CTF form) or to the GAIN domain (cleaved form), respectively. pMPNN (15) was used for this purpose with the “design_only_position” flag enabled and the current GitHub version of the code (https://github.com/dauparas/ProteinMPNN). The following parameters were specified: v_48_020 model weights, backbone noise of 0 Å, and a sampling temperature of 0.1.

In all *in silico* calculations, the amino acid sequences of the GAIN domain and the 7TMD were kept fixed. Only 11 positions within the *Stachel* sequence were allowed to vary, and each of these positions was substituted with all possible proteinogenic amino acids. Depending on the experiment, these simulations yielded 10, 100, 1,000, 10,000, or 100,000 newly designed *Stachel* sequences.

The resulting sequences were clustered based on sequence identity and analyzed for amino acid frequency at each position. An additional approach was applied 7TMD to improve the solubility of the peptide used for experimental activation studies. Here, pMPNN calculations were performed on the synthetic mouse ADGRG2 peptide. The cryo-EM structure of mouse ADGRG2 in complex with the synthetic peptide (PDB: 7WUI (38)) served as the input model, and sequence optimization was carried out on the peptide itself.

Predicted secondary structure propensity of designed *Stachel*-derived peptides was assessed *in silico* from sequence alone. In general, peptides designed for 7TMD binding showed increased α-helical propensity, whereas peptides optimized for GAIN-domain interaction displayed a higher tendency toward extended/β-strand conformations, consistent with the structural constraints imposed during design. A detailed description on the rational design of these peptides is provided in supplementary information (Rational design of additional GPR64/ADGRG2-derived agonistic peptides).

### Generation of aGPCR expression constructs and cell culture

Constructs for functional analyses were generated as previously described by cloning the indicated cDNAs into the mammalian expression vector pcDps (12, 13, 22). Moreover, all aGPCR were N-terminally hemagglutinin (HA) and C-terminally FLAG epitope-tagged. For BRET assays, N-terminally HA-tagged ADGRG2 was amplified using 5′-GGGAGATCTGGTACCGCCACC-3′ and 5′-GGATCCCGGGCCATTTGCTCGATAAAGTG-3′. Primes were used to include a N-terminally *Kpn*I and a C-terminally *Xma*I restriction site. These enzymes were used to insert the receptor into the p-mVenus-N1 (39). All constructs were verified by sequencing. HEK293T, COS-7 and 3T3-L1 cells (ATCC, CRL-3216, and CRL-1651 CL-173) were grown in DMEM supplemented with 10% fetal bovine serum, 100 U/ml penicillin, and 100 μg/ml streptomycin at 37 °C in a humidified incubator with 5% CO_2_.

### *In vitro* functional assay

For second messenger assays, cells were split into 96-well plates (2.0 × 10^4^ cells/well for HEK293T cells, 1.0 × 10^4^ cells/well for COS-7 cells and 1.0 × 10^4^ cells/well for 3T3-L1 cells), whereby the plates for the HEK293T cells had been coated with 0.0002% poly-L-lysine (Merck) in PBS for 30 min at 37 °C prior to use. Cells were transfected with Lipofectamine2000 (Thermo Fisher Scientific) according to manufacturer’s protocol 24 h later. For peptide activation mediated cAMP measurements, 100 ng DNA per well were transfected. 48 h post transfection, cells were washed with serum- and phenol red-free DMEM containing 1 mM 3-isobutyl-methyl-xanthine (IBMX) for 5 min. IBMX inhibits phosphodiesterases and subsequently blocks the degradation of cAMP. To analyze the effects of agonistic peptides (GenScript), transfected cells were treated with 1 mM peptide in DMEM containing 1 mM IBMX for 1 h at 37 °C. To stop the incubation, the media was removed and the cells were lysed with LI buffer (5 mM Hepes (pH 7.6), 0.3% Tween-20, 0.1% BSA, 0.5 mM IBMX) and frozen at −20 °C. To measure cAMP concentrations, samples were thawed and the AlphaScreen cAMP assay kit (PerkinElmer Life Sciences) was applied following the manufacturer’s instructions in 384-well white OptiPlate microplates (PerkinElmer Life Sciences) utilizing the Envsion multimode plate reader (PerkinElmer Life Sciences).

Measurements of IP1 were performed in HEK293T cells with the IP-One Tb kit (Cisbio) according to the manufacturer’s protocol. Here, IP1 accumulation is achieved by 50 mM LiCl contained in the stimulation buffer which blocks the degradation of IP1 to myo-inositol. After 48 h of transfection, HEK293T cells were incubated with IP1 stimulation buffer containing 1 mM peptide or respective controls. After 30 min cells were lysed. Accumulated IP1 was determined in cell extracts using ProxiPlate-384 Plus microplates (PerkinElmer) with the EnVision Multilabel Reader (PerkinElmer).

BRET assays were performed in white 96-well plates (Greiner). Thereto, HA-ADGRG2-Venus and Gs-Rluc were co-transfected in a ratio 10:1. The baseline (Hank’s balanced salt solution [HBSS] containing Rluc substrate) was recorded for 5 min, the HBSS removed and replaced by peptide containing HBSS-Rluc substrate. Stimulation was determined as area under the curve after baseline normalization (39). Cell surface expression level determination was performed as described previously.

For siRNA-mediated knockdown in 3T3-L1 cells, 20 μl OptiMEM (Thermo Fisher Scientific) were mixed with 0.2 μl RNAiMAX (Thermo Fisher Scientific) and 1 pmol siRNA (Origene) per well. After an incubation for 20 min at 37 °C, 0.9 × 10^4^ cells in 50 μl OptiMEM were added per well. DMEM was added after 5 h. The siRNAs used were from Origene Technologies (https://www.origene.com/catalog/rnai/sirna-oligo-duplexes/sr421606-adgrg2-mouse-sirna-oligo-duplex-locus-id-237175). The mouse Adgrg2-specific siRNA was rGrArArGrArGrGrUrUrUrUrArCrUrGrArUrArUrArCrArAGA and the control was duplex siRNA (rCrGrUrUrArArUrCrGrCrGrUrArUrArArUrArCrGrCrGrUAT/rArUrArCrGrCrGrUrArUrUrArUrArCrGrCrGrArUrUrArArCrGrArC).

### Collection and reprocessing of RNA sequencing data

Publicly available RNA-seq data of HEK293T and COS-7 cells were collected from the NCBI GEO database (HEK293T: GSE285962; COS-7: GSE121304). Only control lipofected and control treated samples were chosen for analysis (HEK293T: GSM8713924, GSM8713925, GSM8713926, and GSM8713927; COS-7: GSM3431118, GSM3431119, and GSM3431120). Reprocessing of raw sequencing data in fastq format was performed using kallisto version 0.46.1. The reference genomes of *Homo sapiens* and *Chlorocebus sabaeus* and their respective annotation were obtained from Ensembl (v115).

## Data availability

All the data are included in the main text or supporting information.

## Supporting information

This article contains supporting information (1, 11, 17, 18, 22, 39, 41, 42).

## Conflict of interest

The authors declare that they have no conflicts of interest with the contents of this article.
