# Supplementary material for: Evolution- and structure-guided redesign of the Stachel motif yields soluble and potent modulators of adhesion GPCRs
Source: J Biol Chem. 2026 Jul 13;302(9):113323. doi: 10.1016/j.jbc.2026.113323 (PMC13470171; doi:10.1016/j.jbc.2026.113323)
Supplement: Supplementary file 1 [file mmc1.pdf]

Supplementary Information

Evolution- and structure-guided redesign of the *Stachel* motif yields soluble and potent modulators of adhesion GPCRs

Abibe Useini[1], Yue Feng[2], Isabell Kaczmarek[2,3], Georg Künze[1,4,5], Doreen Thor[2], Torsten Schöneberg[2,6,*]

[1]Institute for Drug Discovery, Leipzig University, Brüderstraße 34, 04103 Leipzig, Germany
[2]Rudolf Schönheimer Institute of Biochemistry, Molecular Biochemistry, Medical Faculty, University of Leipzig, Johannisallee 30, 04103 Leipzig, Germany
[3]Department of Pharmacy, Institute of Pharmaceutical Chemistry, University of Marburg, Marburg, Germany
[4]Interdisciplinary Center for Bioinformatics, Leipzig University, 04107 Leipzig, Germany
[5]Center for Scalable Data Analytics and Artificial Intelligence, Leipzig University, 04105 Leipzig, Germany
[6]School of Medicine, University of Global Health Equity, Kigali, Rwanda

*To whom correspondences should be addressed:
Torsten Schöneberg, schoberg@medizin.uni-leipzig.de

A

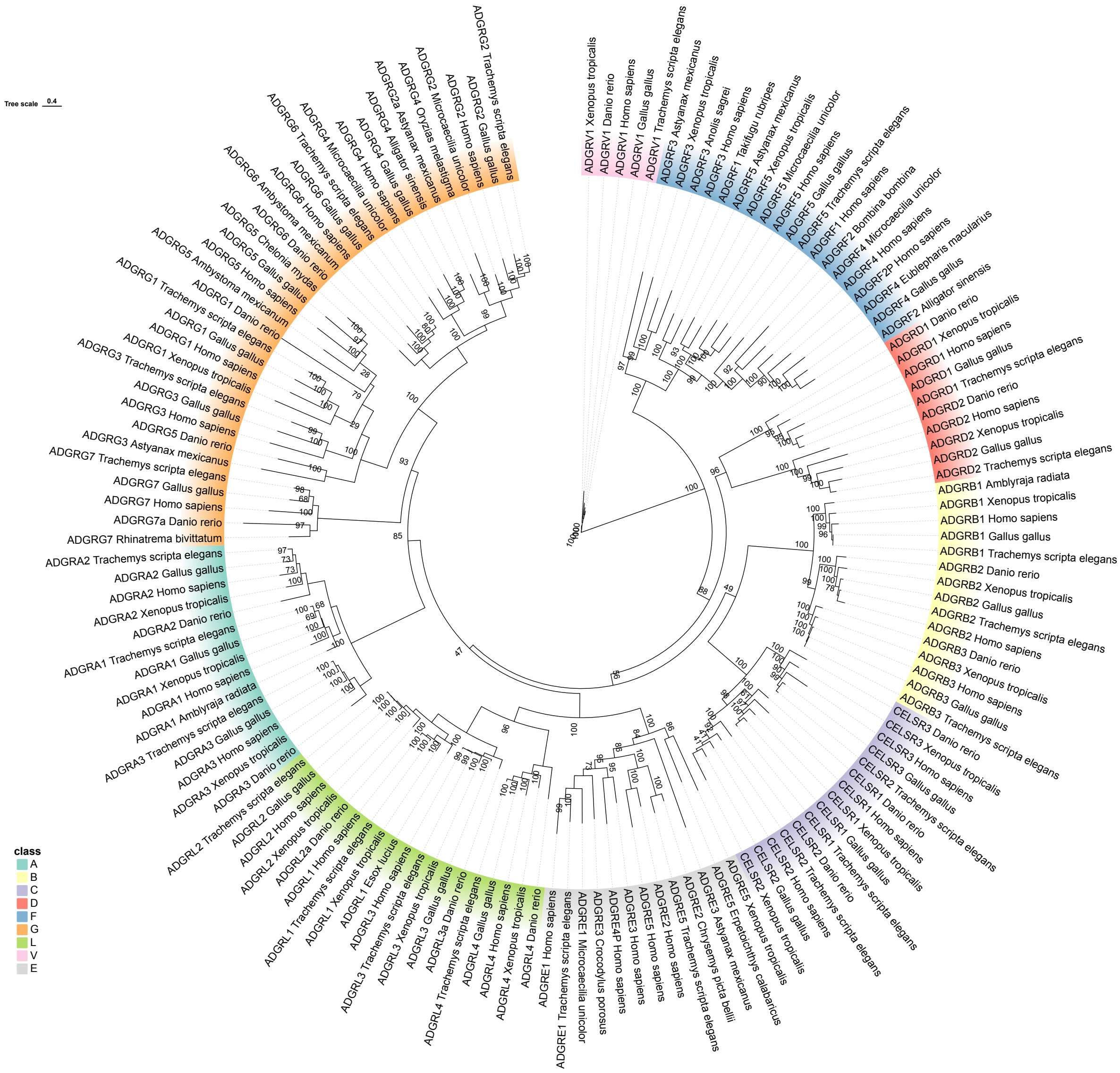
Tree scale 0.4
class
A
B
C
D
F
G
L
V
E
ADGRV1 Xenopus tropicalis
ADGRV1 Danio rerio
ADGRV1 Homo sapiens
ADGRV1 Gallus gallus
ADGRV1 Trachemys scripta elegans
ADGRF3 Astyanax mexicanus
ADGRF3 Xenopus tropicalis
ADGRF3 Anolis sagrei
ADGRF3 Homo sapiens
ADGRF1 Takifugu rubripes
ADGRF5 Astyanax mexicanus
ADGRF5 Xenopus tropicalis
ADGRF5 Microcaecilia unicolor
ADGRF5 Homo sapiens
ADGRF5 Gallus gallus
ADGRF5 Trachemys scripta elegans
ADGRF1 Homo sapiens
ADGRF2 Bombina bombina
ADGRF4 Microcaecilia unicolor
ADGRF4 Homo sapiens
ADGRF2P Homo sapiens
ADGRF4 Eublepharis macularius
ADGRF4 Gallus gallus
ADGRF2 Alligator sinensis
ADGRD1 Danio rerio
ADGRD1 Xenopus tropicalis
ADGRD1 Homo sapiens
ADGRD1 Gallus gallus
ADGRD1 Trachemys scripta elegans
ADGRD2 Danio rerio
ADGRD2 Homo sapiens
ADGRD2 Xenopus tropicalis
ADGRD2 Gallus gallus
ADGRD2 Trachemys scripta elegans
ADGRB1 Amblyraja radiata
ADGRB1 Xenopus tropicalis
ADGRB1 Homo sapiens
ADGRB1 Gallus gallus
ADGRB1 Trachemys scripta elegans
ADGRB2 Danio rerio
ADGRB2 Xenopus tropicalis
ADGRB2 Gallus gallus
ADGRB2 Trachemys scripta elegans
ADGRB2 Homo sapiens
ADGRB3 Danio rerio
ADGRB3 Xenopus tropicalis
ADGRB3 Homo sapiens
ADGRB3 Gallus gallus
ADGRB3 Trachemys scripta elegans
CELSR3 Danio rerio
CELSR3 Xenopus tropicalis
CELSR3 Homo sapiens
CELSR3 Gallus gallus
CELSR2 Trachemys scripta elegans
CELSR1 Danio rerio
CELSR1 Homo sapiens
CELSR1 Xenopus tropicalis
CELSR1 Gallus gallus
CELSR1 Trachemys scripta elegans
CELSR2 Danio rerio
CELSR2 Homo sapiens
CELSR2 Trachemys scripta elegans
CELSR2 Gallus gallus
CELSR2 Xenopus tropicalis
ADGRE5 Xenopus tropicalis
ADGRE5 Erpetoichthys calabaricus
ADGRE3 Astyanax mexicanus
ADGRE2 Chrysemys picta bellii
ADGRE5 Trachemys scripta elegans
ADGRE2 Homo sapiens
ADGRE5 Homo sapiens
ADGRE3 Homo sapiens
ADGRE4P Homo sapiens
ADGRE3 Crocodylus porosus
ADGRE1 Microcaecilia unicolor
ADGRE1 Trachemys scripta elegans
ADGRE1 Homo sapiens
ADGRL4 Danio rerio
ADGRL4 Xenopus tropicalis
ADGRL4 Homo sapiens
ADGRL4 Gallus gallus
ADGRL4 Trachemys scripta elegans
ADGRL3a Danio rerio
ADGRL3 Gallus gallus
ADGRL3 Xenopus tropicalis
ADGRL3 Trachemys scripta elegans
ADGRL3 Homo sapiens
ADGRL1 Esox lucius
ADGRL1 Xenopus tropicalis
ADGRL1 Trachemys scripta elegans
ADGRL1 Homo sapiens
ADGRL2a Danio rerio
ADGRL2 Xenopus tropicalis
ADGRL2 Homo sapiens
ADGRL2 Gallus gallus
ADGRL2 Trachemys scripta elegans
ADGRA3 Danio rerio
ADGRA3 Xenopus tropicalis
ADGRA3 Homo sapiens
ADGRA3 Gallus gallus
ADGRA3 Trachemys scripta elegans
ADGRA1 Amblyraja radiata
ADGRA1 Homo sapiens
ADGRA1 Xenopus tropicalis
ADGRA1 Gallus gallus
ADGRA1 Trachemys scripta elegans
ADGRA2 Danio rerio
ADGRA2 Xenopus tropicalis
ADGRA2 Homo sapiens
ADGRA2 Gallus gallus
ADGRA2 Trachemys scripta elegans
ADGRG7 Rhinatrema bivittatum
ADGRG7a Danio rerio
ADGRG7 Homo sapiens
ADGRG7 Gallus gallus
ADGRG7 Trachemys scripta elegans
ADGRG3 Astyanax mexicanus
ADGRG5 Danio rerio
ADGRG3 Homo sapiens
ADGRG3 Gallus gallus
ADGRG3 Trachemys scripta elegans
ADGRG1 Xenopus tropicalis
ADGRG1 Homo sapiens
ADGRG1 Gallus gallus
ADGRG1 Trachemys scripta elegans
ADGRG1 Danio rerio
ADGRG5 Homo sapiens
ADGRG5 Ambystoma mexicanum
ADGRG5 Gallus gallus
ADGRG6 Chelonia mydas
ADGRG6 Danio rerio
ADGRG6 Ambystoma mexicanum
ADGRG6 Homo sapiens
ADGRG6 Gallus gallus
ADGRG6 Trachemys scripta elegans
ADGRG4 Microcaecilia unicolor
ADGRG4 Homo sapiens
ADGRG4 Gallus gallus
ADGRG4 Alligator sinensis
ADGRG2a Astyanax mexicanus
ADGRG4 Oryzias melastigma
ADGRG2 Microcaecilia unicolor
ADGRG2 Homo sapiens
ADGRG2 Gallus gallus
ADGRG2 Trachemys scripta elegans

B

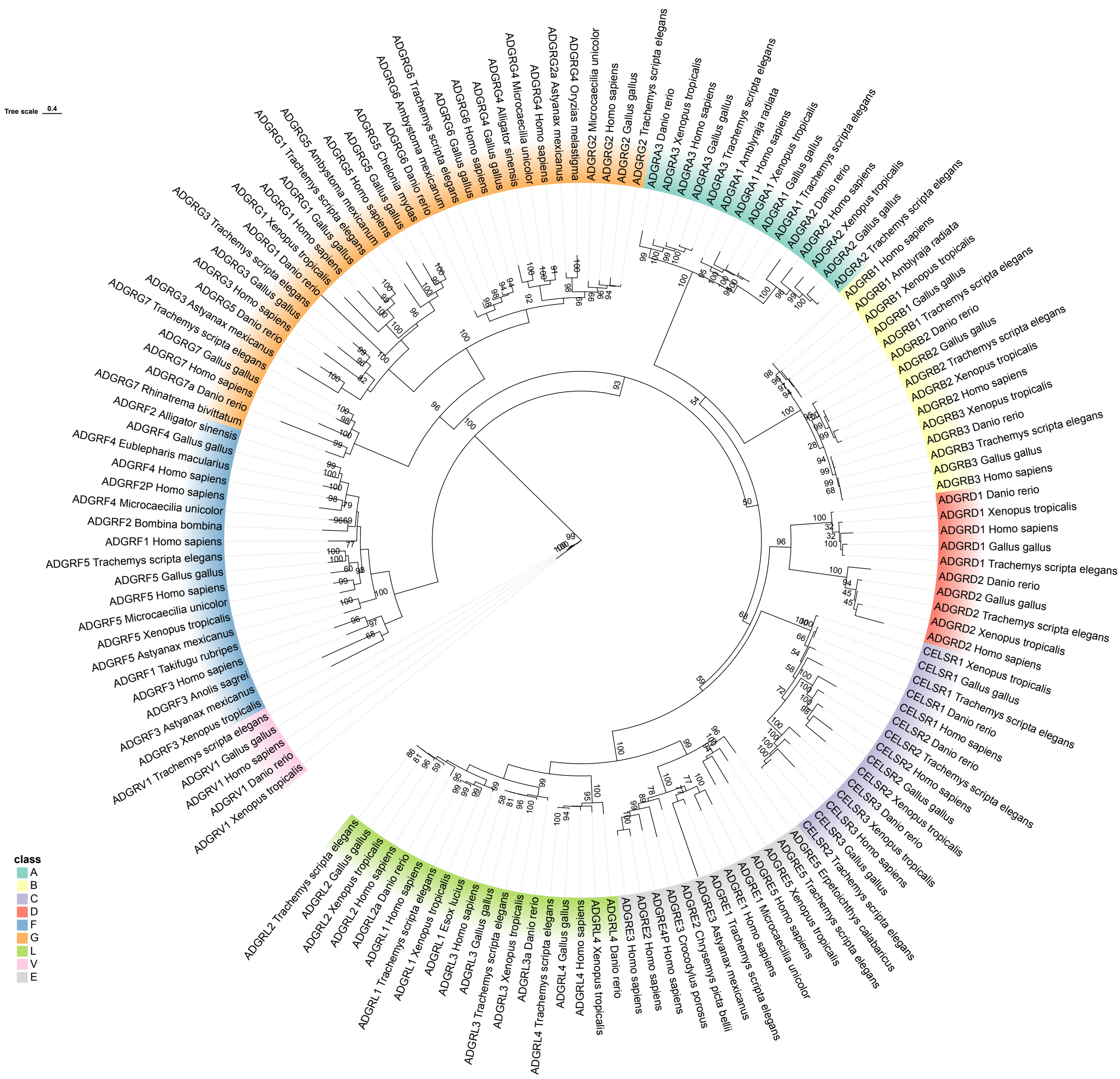
Tree scale 0.4
class
A
B
C
D
F
G
L
V
E
ADGRG6 Trachemys scripta elegans
ADGRG6 Ambystoma mexicanum
ADGRG6 Danio rerio
ADGRG6 Gallus gallus
ADGRG6 Homo sapiens
ADGRG4 Gallus gallus
ADGRG4 Alligator sinensis
ADGRG4 Microcaecilia unicolor
ADGRG4 Homo sapiens
ADGRG2a Astyanax mexicanus
ADGRG4 Oryzias melastigma
ADGRG2 Microcaecilia unicolor
ADGRG2 Homo sapiens
ADGRG2 Gallus gallus
ADGRG2 Trachemys scripta elegans
ADGRA3 Danio rerio
ADGRA3 Xenopus tropicalis
ADGRA3 Homo sapiens
ADGRA3 Gallus gallus
ADGRA3 Trachemys scripta elegans
ADGRA1 Amblyraja radiata
ADGRA1 Homo sapiens
ADGRA1 Xenopus tropicalis
ADGRA1 Gallus gallus
ADGRA1 Trachemys scripta elegans
ADGRA2 Danio rerio
ADGRA2 Homo sapiens
ADGRA2 Xenopus tropicalis
ADGRA2 Gallus gallus
ADGRA2 Trachemys scripta elegans
ADGRB1 Homo sapiens
ADGRB1 Amblyraja radiata
ADGRB1 Xenopus tropicalis
ADGRB1 Gallus gallus
ADGRB1 Trachemys scripta elegans
ADGRB2 Danio rerio
ADGRB2 Gallus gallus
ADGRB2 Trachemys scripta elegans
ADGRB2 Xenopus tropicalis
ADGRB2 Homo sapiens
ADGRB3 Xenopus tropicalis
ADGRB3 Danio rerio
ADGRB3 Trachemys scripta elegans
ADGRB3 Gallus gallus
ADGRB3 Homo sapiens
ADGRD1 Danio rerio
ADGRD1 Xenopus tropicalis
ADGRD1 Homo sapiens
ADGRD1 Gallus gallus
ADGRD1 Trachemys scripta elegans
ADGRD2 Danio rerio
ADGRD2 Gallus gallus
ADGRD2 Trachemys scripta elegans
ADGRD2 Xenopus tropicalis
ADGRD2 Homo sapiens
CELSR1 Xenopus tropicalis
CELSR1 Gallus gallus
CELSR1 Trachemys scripta elegans
CELSR1 Danio rerio
CELSR1 Homo sapiens
CELSR2 Danio rerio
CELSR2 Trachemys scripta elegans
CELSR2 Homo sapiens
CELSR2 Gallus gallus
CELSR2 Xenopus tropicalis
CELSR3 Danio rerio
CELSR3 Xenopus tropicalis
CELSR3 Homo sapiens
CELSR3 Gallus gallus
CELSR3 Trachemys scripta elegans
ADGRE5 Erpetoichthys calabaricus
ADGRE5 Trachemys scripta elegans
ADGRE5 Xenopus tropicalis
ADGRE5 Homo sapiens
ADGRE1 Microcaecilia unicolor
ADGRE1 Homo sapiens
ADGRE1 Trachemys scripta elegans
ADGRE3 Astyanax mexicanus
ADGRE2 Chrysemys picta bellii
ADGRE3 Crocodylus porosus
ADGRE4P Homo sapiens
ADGRE2 Homo sapiens
ADGRE3 Homo sapiens
ADGRL4 Danio rerio
ADGRL4 Xenopus tropicalis
ADGRL4 Homo sapiens
ADGRL4 Gallus gallus
ADGRL4 Trachemys scripta elegans
ADGRL3a Danio rerio
ADGRL3 Xenopus tropicalis
ADGRL3 Trachemys scripta elegans
ADGRL3 Gallus gallus
ADGRL3 Homo sapiens
ADGRL1 Esox lucius
ADGRL1 Xenopus tropicalis
ADGRL1 Trachemys scripta elegans
ADGRL1 Homo sapiens
ADGRL2a Danio rerio
ADGRL2 Homo sapiens
ADGRL2 Xenopus tropicalis
ADGRL2 Gallus gallus
ADGRL2 Trachemys scripta elegans
ADGRV1 Xenopus tropicalis
ADGRV1 Danio rerio
ADGRV1 Homo sapiens
ADGRV1 Gallus gallus
ADGRV1 Trachemys scripta elegans
ADGRF3 Xenopus tropicalis
ADGRF3 Astyanax mexicanus
ADGRF3 Anolis sagrei
ADGRF3 Homo sapiens
ADGRF1 Takifugu rubripes
ADGRF5 Astyanax mexicanus
ADGRF5 Xenopus tropicalis
ADGRF5 Microcaecilia unicolor
ADGRF5 Homo sapiens
ADGRF5 Gallus gallus
ADGRF5 Trachemys scripta elegans
ADGRF1 Homo sapiens
ADGRF2 Bombina bombina
ADGRF4 Microcaecilia unicolor
ADGRF2P Homo sapiens
ADGRF4 Homo sapiens
ADGRF4 Eublepharis macularius
ADGRF4 Gallus gallus
ADGRF2 Alligator sinensis
ADGRG7 Rhinatrema bivittatum
ADGRG7a Danio rerio
ADGRG7 Homo sapiens
ADGRG7 Gallus gallus
ADGRG7 Trachemys scripta elegans
ADGRG3 Astyanax mexicanus
ADGRG5 Danio rerio
ADGRG3 Homo sapiens
ADGRG3 Gallus gallus
ADGRG3 Trachemys scripta elegans
ADGRG1 Danio rerio
ADGRG1 Xenopus tropicalis
ADGRG1 Homo sapiens
ADGRG1 Gallus gallus
ADGRG1 Trachemys scripta elegans
ADGRG5 Ambystoma mexicanum
ADGRG5 Homo sapiens
ADGRG5 Gallus gallus
ADGRG5 Chelonia mydas

C

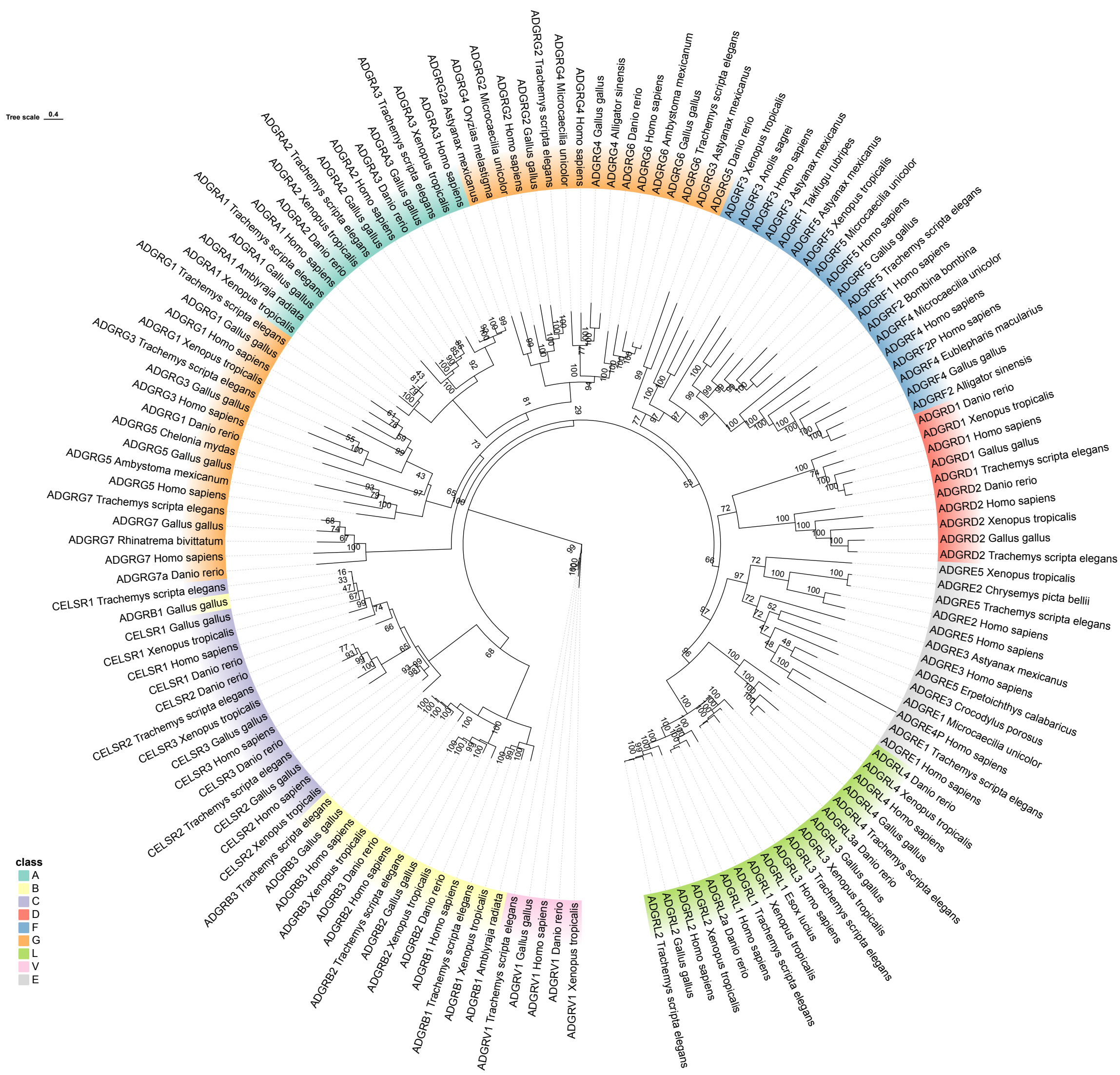

Suppl. Figure S1. The coevolution of the GAIN and 7TM domains. To accurately assign the amino acid sequences of the GAIN plus 7TMD (**A**), the 7TMD alone (**B**), and the GAIN domain alone (**C**) to different aGPCR groups, representatives from all aGPCR families across four vertebrate classes were included in the analysis. Sequence alignments of the indicated domains were used to construct phylogenetic trees with the maximum likelihood method and 10,000 bootstraps, with ADGRV orthologs defined as the outgroup. In the condensed triangle projections of the trees, different groups are color-coded, and bootstrap values >75 are considered significant.

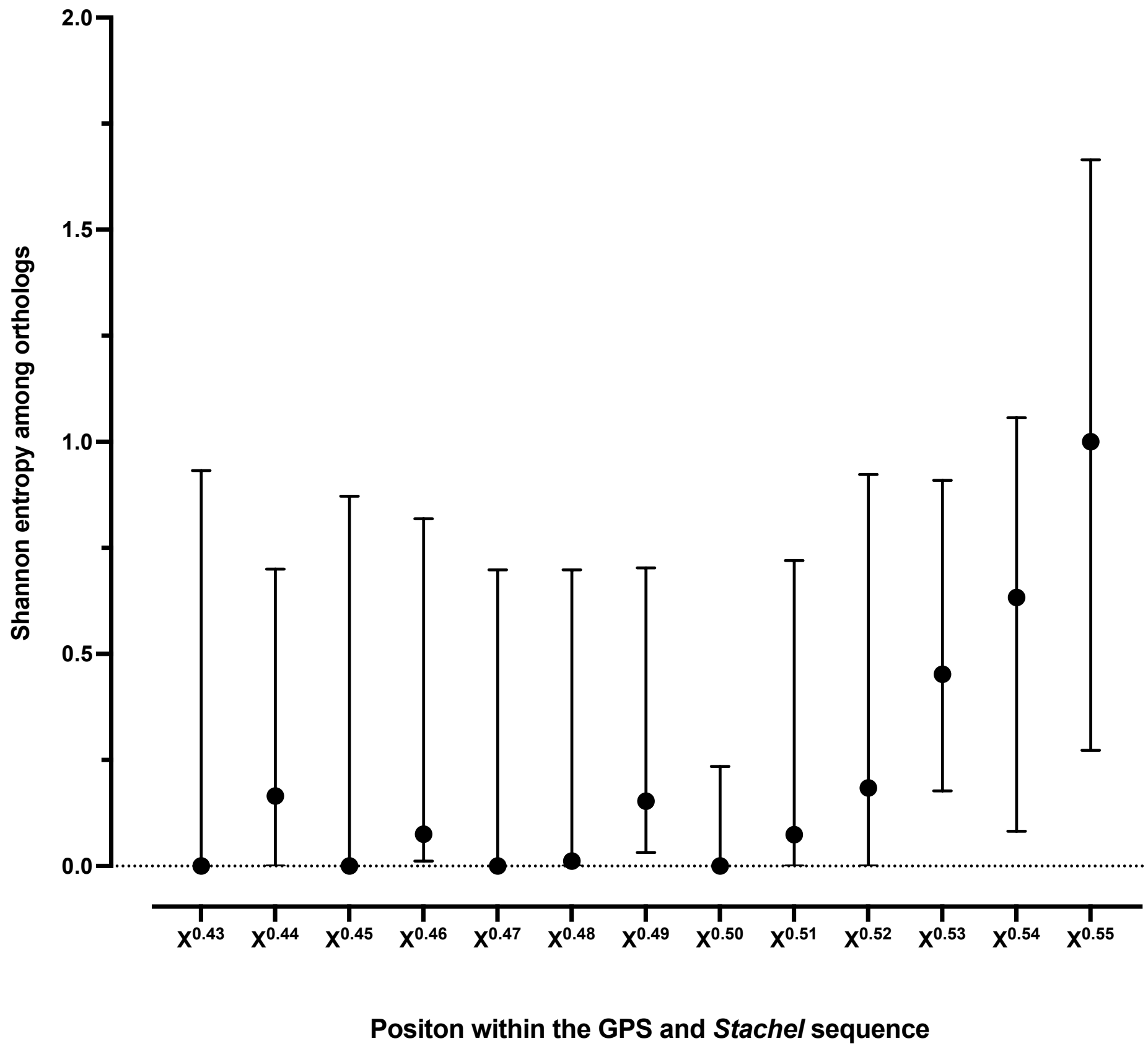

Suppl. Figure S2. The conservation of the GPS and **Stachel** ***sequences in subsampled vertebrate aGPCR.*** Orthologous amino acid sequences of vertebrate aGPCRs (13,611 sequences) were assigned to the 33 mammalian aGPCR members and aligned. From 11 (A2, B1, C1, D1, E1, F5, G1, G2, G7, L1, V1) of the 33 alignments, the positional entropy within the GPS and *Stachel* regions was calculated. Because G group is actually phylogenetically the most diverse with 3 subgroups (G1/G3/G5, G2/G4/G6, G7) (1, 2) one member of each was included. Residue numbering follows the system described in reference (3). "X" denotes individual residue positions, and the numbering indicates the distance of each position from the most conserved residue among all vertebrate aGPCRs analyzed ($X^{0.50}$). The median entropy values and their 95% confidence intervals are shown.

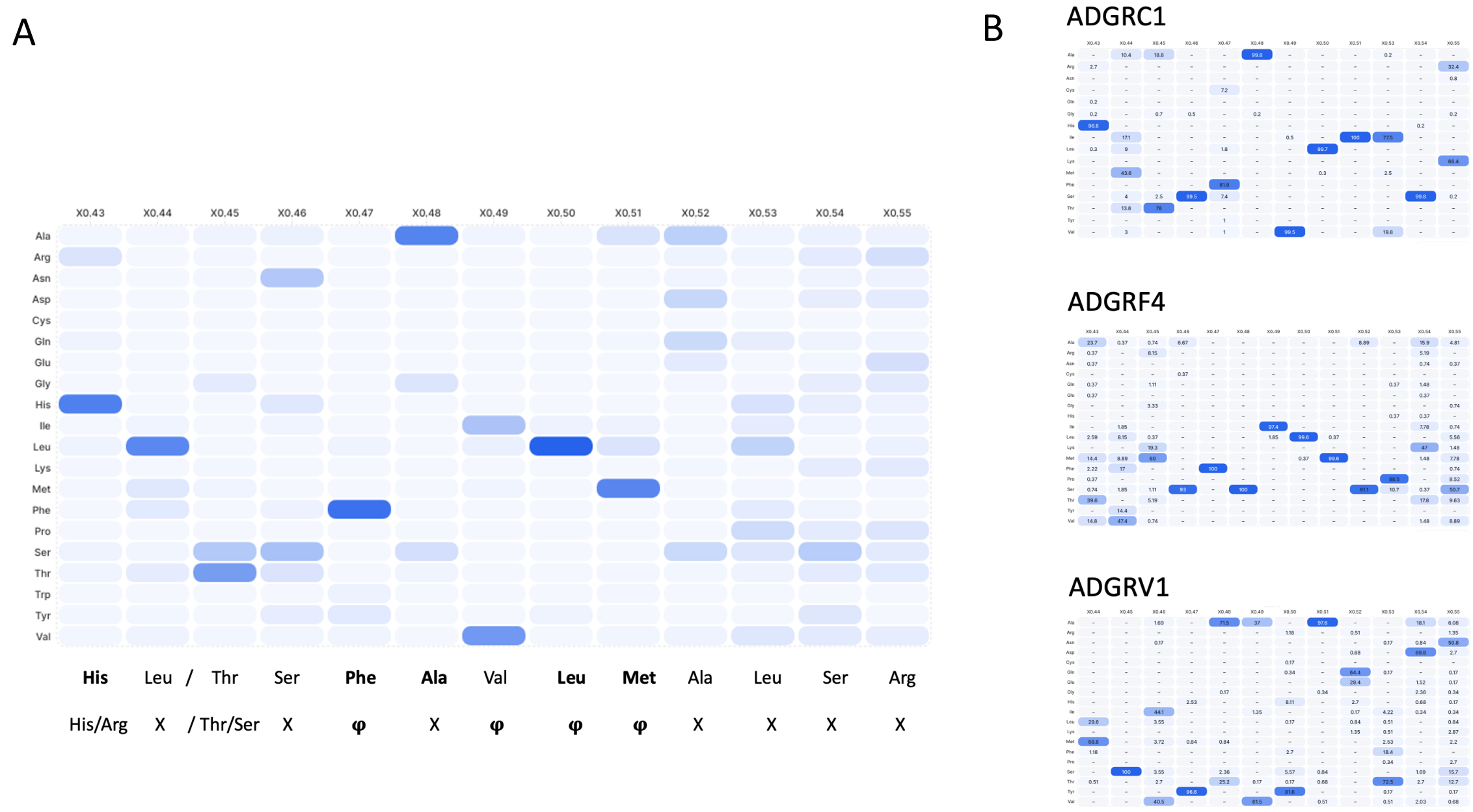

Suppl. Figure S3. Consensus of the **Stachel** ***sequence and comparison with outliers.*** The average frequencies of amino acid residues at each position of the GPS/*Stachel* sequences across all aGPCR members are shown in a heatmap (**A**). The consensus sequence (bottom of the heatmap) represents the most frequent amino acid at each position. Residues shown in bold have a frequency >70% across all aGPCRs. As shown in Figure 3, most aGPCR members cluster around ADGRC1 (C1) in the center. ADGRC1 (**B**, upper right) approximately represents the average frequencies (left panel). Members of the F (e.g., ADGRF4) and V (ADGRV1) groups display amino acid residue patterns distinct from the average pattern of the other aGPCR members. Heatmaps were generated using https://visualize.graphy.app.

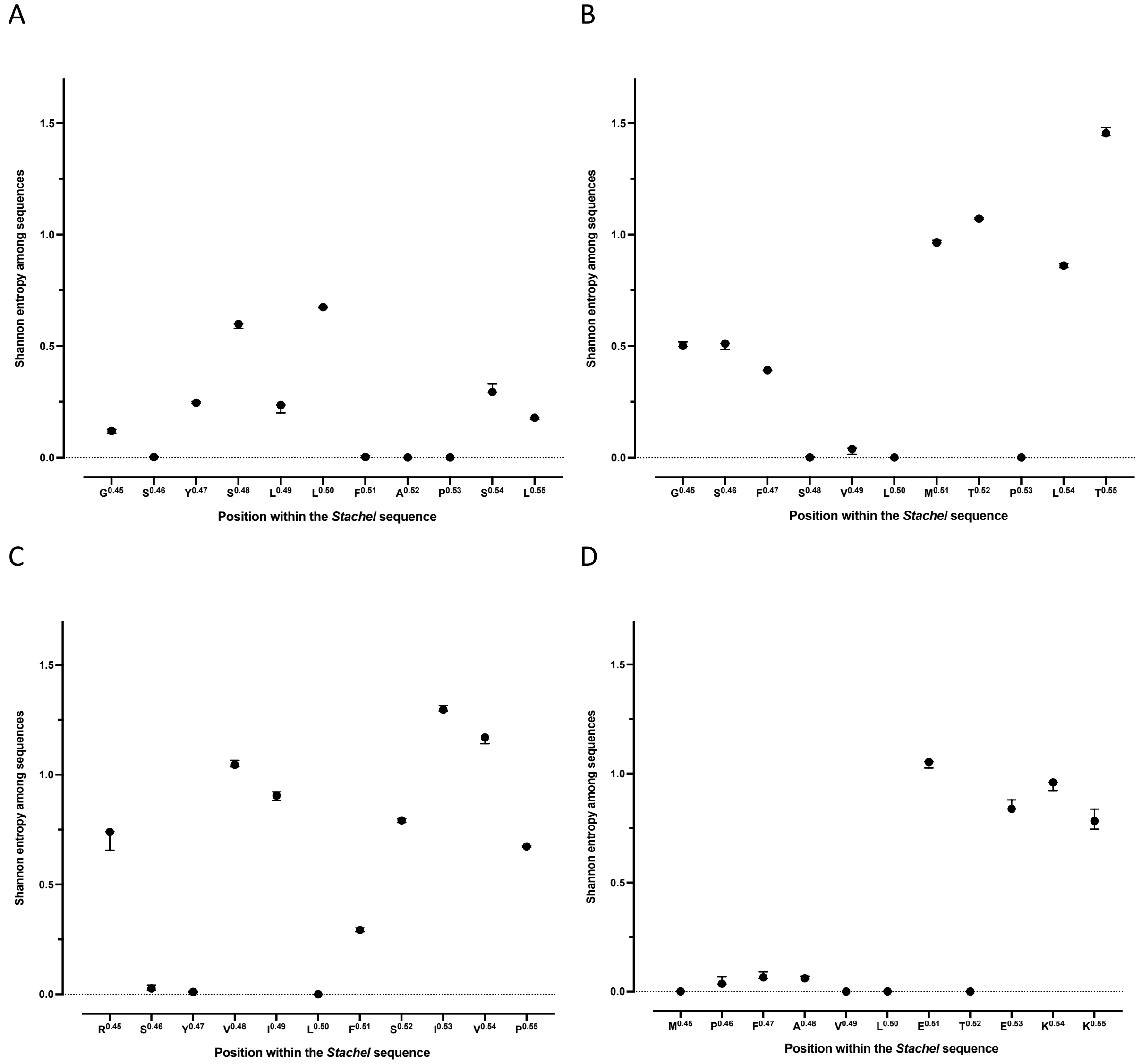

Suppl. Figure S4. The entropy of the* Stachel *sequence variants of ADGRF1 and ADGRL3 generated with ProteinMPNN. Amino acid sequences of the *Stachel* of ADGRF1 (TSFSILMSPFV) (**A, B**) and ADGRL3 (TNFAVLMAHVE) (**C, D**) which were found to bind to the 7TMD (**A, C**) and GAIN domain (cleaved) (**B, D**) were aligned (ProteinMPNN data from 1000, 10,000 and 100,000 iterations). From the alignments, the entropy of every position within the *Stachel* sequences was calculated. The consensus sequences are given at the X axes and the 7TMD and the GAIN domain differently restrict the *Stachel* sequence in the *in-silico Stachel* simulation. The median and the 95% confidence interval are shown.

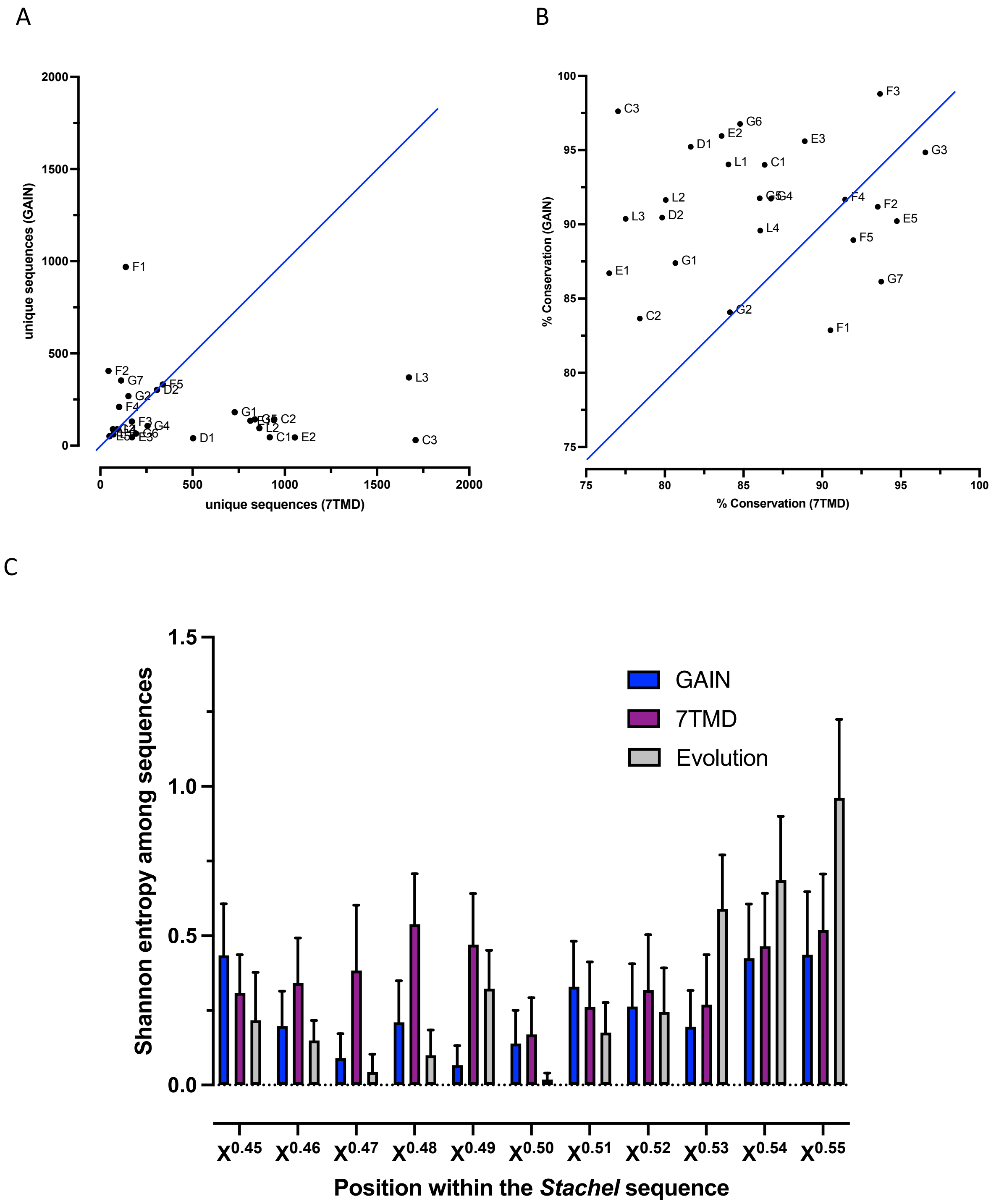

Suppl. Figure S5. 7TMD and GAIN domain constrain the **Stachel** ***sequences of aGPCR.*** ProteinMPNN was used to generate amino acid sequences of the *Stachel* of 25 aGPCR binding to the 7TMD and GAIN domain separately. The results were analyzed in respect to the number of unique sequences (**A**) and the average conservation of the consensus *Stachel* sequences (**B**) bound to the 7TMD and GAIN domain. The consensus sequences were different between the 7TMD- and the GAIN domain-restricted *in silico* analysis. The blue lines indicate the 1:1 ratio between the X-Y axes. (**C**) The positional Shannon entropy was determined and compared between the 7TMD and GAIN domain and for comparison the evolutionary constraint for the 25 included aGPCR *Stachel* sequences are given (grey bars). Unique amino acid sequences of the *Stachel* of human 25 aGPCR members which were found to bind to the 7TMD and GAIN domain (cleaved) were aligned (data from 10,000 iterations). From the alignments, the entropy of every position within the *Stachel* sequences was calculated. The median and the 95% confidence interval are shown. Only the entropies of GAIN-restricted positions are significantly lower (p=0.04) than those of the 7TMD-restricted positions.

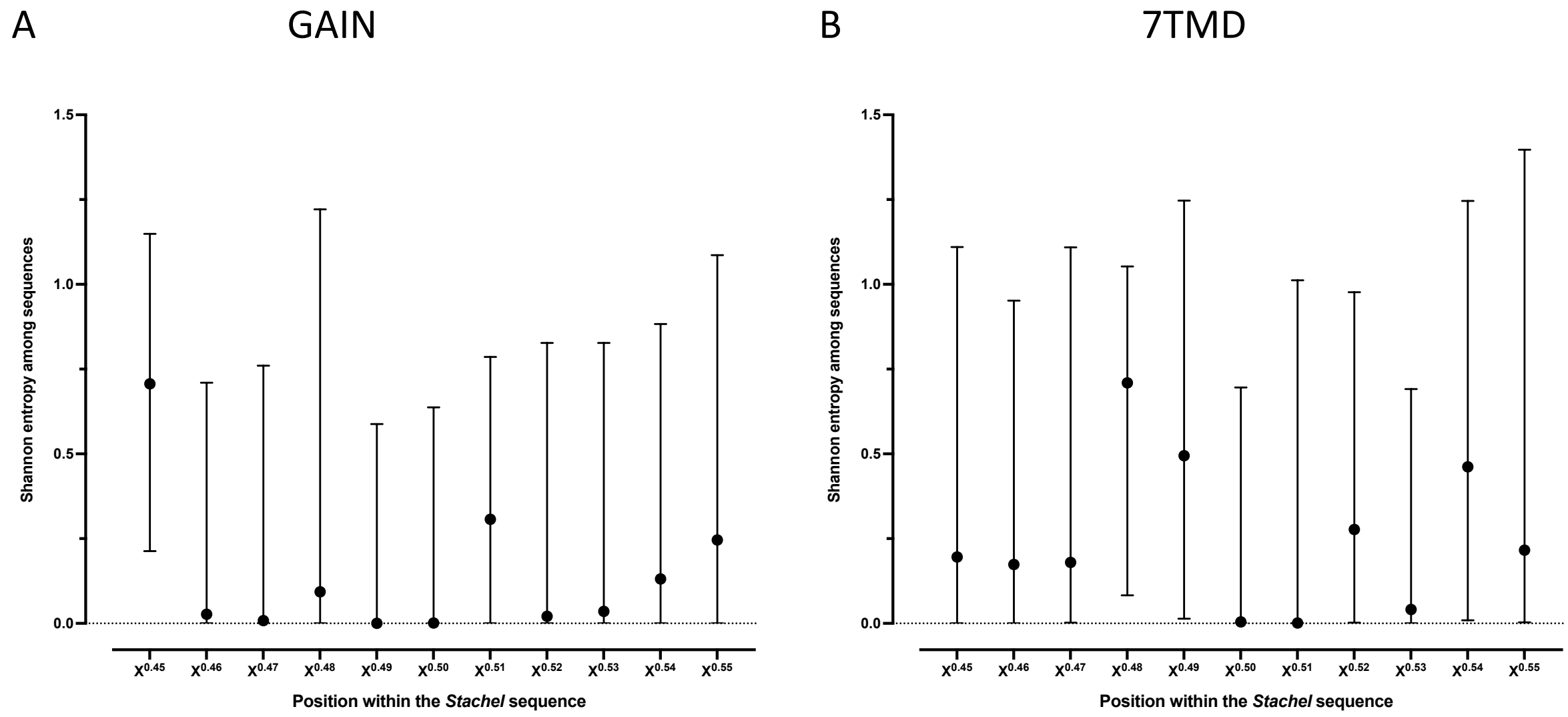

Suppl. Figure S6. The entropy of a* Stachel *sequence variant subset in in-silico mutated and tested aGPCRs. Amino acid sequences of the *Stachel* of 8 analyzed aGPCRs (C1, D1, E1, F5, G1, G2, G7, L1) which were found to bind to the GAIN domain (cleaved) (**A**) and the 7TMD (**B**) were aligned (data from 10,000 iterations). From the alignments, the entropy of every position within the *Stachel* sequence variants was calculated. The consensus sequences were different between the 7TMD and the GAIN domain restricted *in silico*-tested *Stachel* sequence is given at the X axes. The median and the 95% confidence interval are shown.

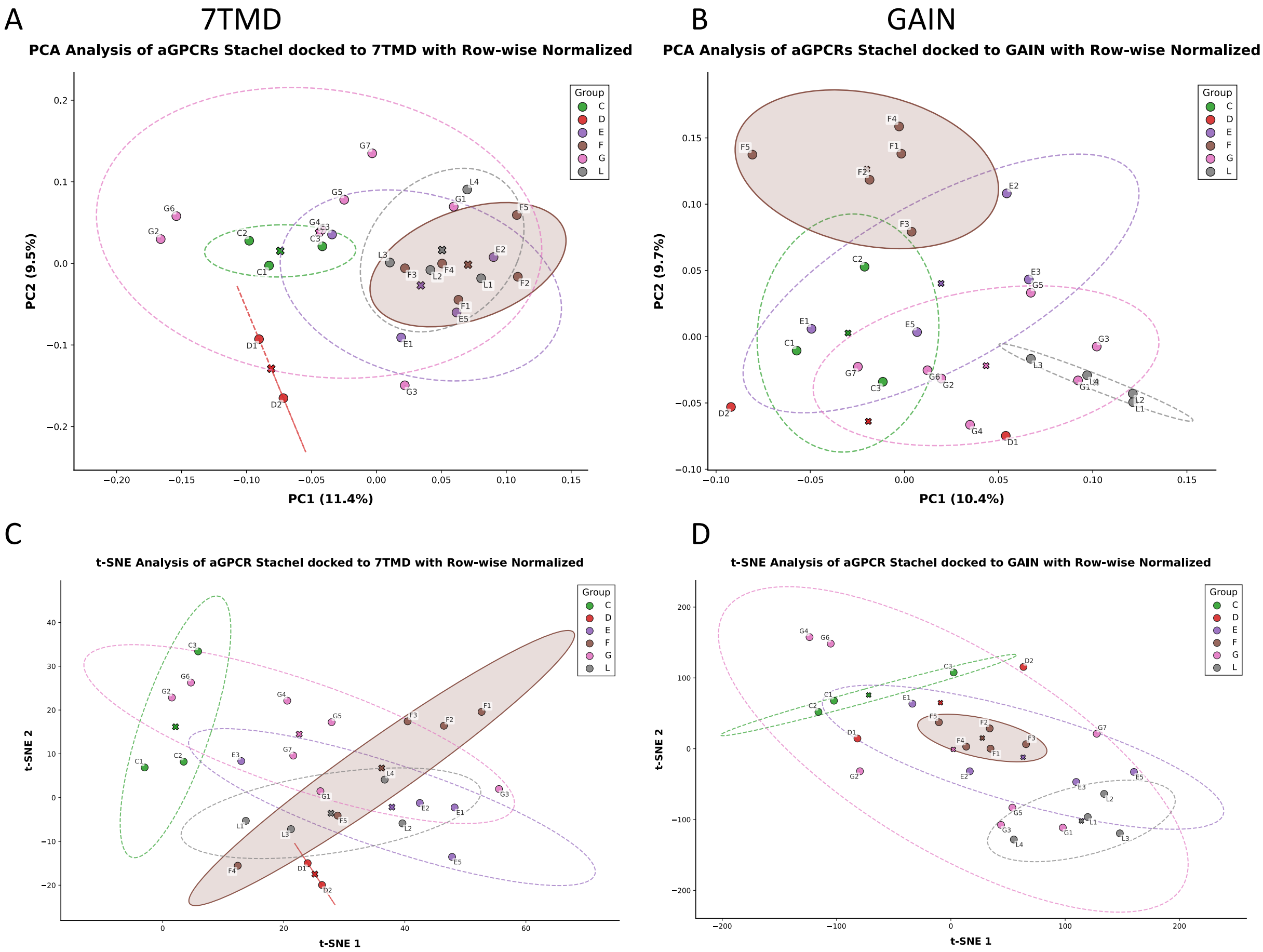

Suppl. Figure S7. PCA and t-SNE analysis of the amino acid residue frequency at their positions in the* Stachel *sequences generated by simulation. To compare amino acid substitution patterns among aGPCR members, the *Stachel* sequence simulation results were aligned, and the amino acid residue frequency was determined at each position in the *Stachel* sequences to the 7TMD (**A, C**) and the GAIN domain (**B, D**). The principal component analysis (PCA) and the t-SNE analysis integrated four parameters: aGPCR member, position within the *Stachel*, amino acid residue, and residue frequency. (**A, B**) The PCA of these parameters is shown for each aGPCR member. (**C, D**) The t-SNE analysis of these parameters is shown for each aGPCR member. Clusters of the paralog group F are marked with solid circles showing some overlap with E and L group members in the *Stachel*-7TMD analyses but not in the *Stachel*-GAIN analyses. Areas occupied by other group members are shown with dashed circles.

A

Ortholog consensus	G2	TSFGVLLDLSR
human	G2	TSFGVLLDLSR
mouse	G2	TSFG**I**LLDLSR
p64-wt-long	G2	TSFGVLLDLSRTSLP
p64-wt	G2	TSFGVLLDLSR
p64-1 (consensus)	G2	**P**SF**T**VLLDLSR
p64-2 (frequent)	G2	**P**S**YT**VLLDLSR

B

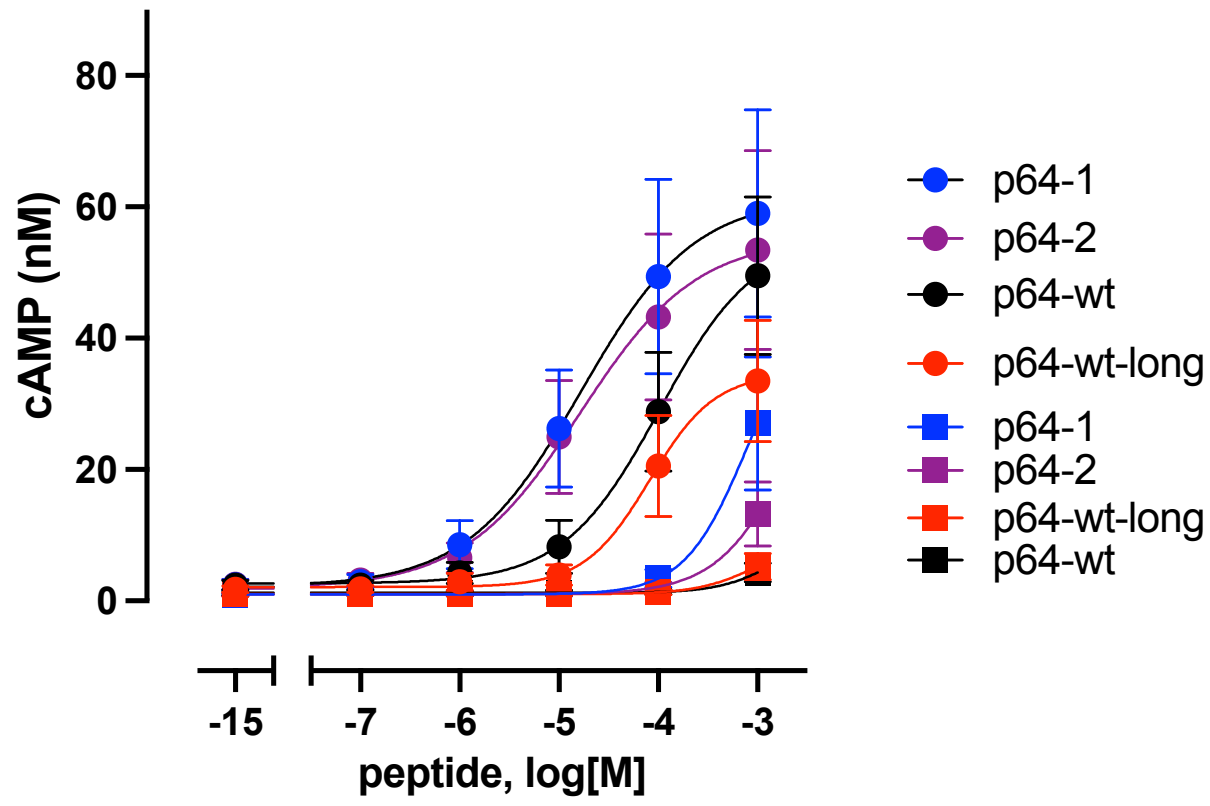

Suppl. Figure S8. Functional analysis of ADGRG2-Stachel sequence variants generated by simulation. (**A**) Alignment of 446 vertebrate orthologs revealed an ortholog consensus sequence identical to the human sequence. Mouse ADGRG2 differs only at position $X^{0.49}$ (V to I), where 81.8% of the investigated orthologs contain valine and 18.2% contain isoleucine. Previous work identified *Stachel* sequence-derived peptides as agonists at the mouse ADGRG2 (4). AlphaFold structure of human ADGRG2 was obtained and *Stachel* sequence simulations using pMPNN were performed, and peptides p64-1 and p64-2 were synthesized based on the consensus sequence and a highly frequent designed sequence. (**B**) COS-7 cells were transiently transfected with mouse ADGRG2, and the indicated peptides were tested in cAMP assays (circles). For control purposes, all peptides were also tested in empty-vector (pcDps)–transfected cells (squares). cAMP levels as a function of peptide concentration are shown. Data are presented as mean ± S.E.M. from 4-5 independent experiments performed with technical replicates.

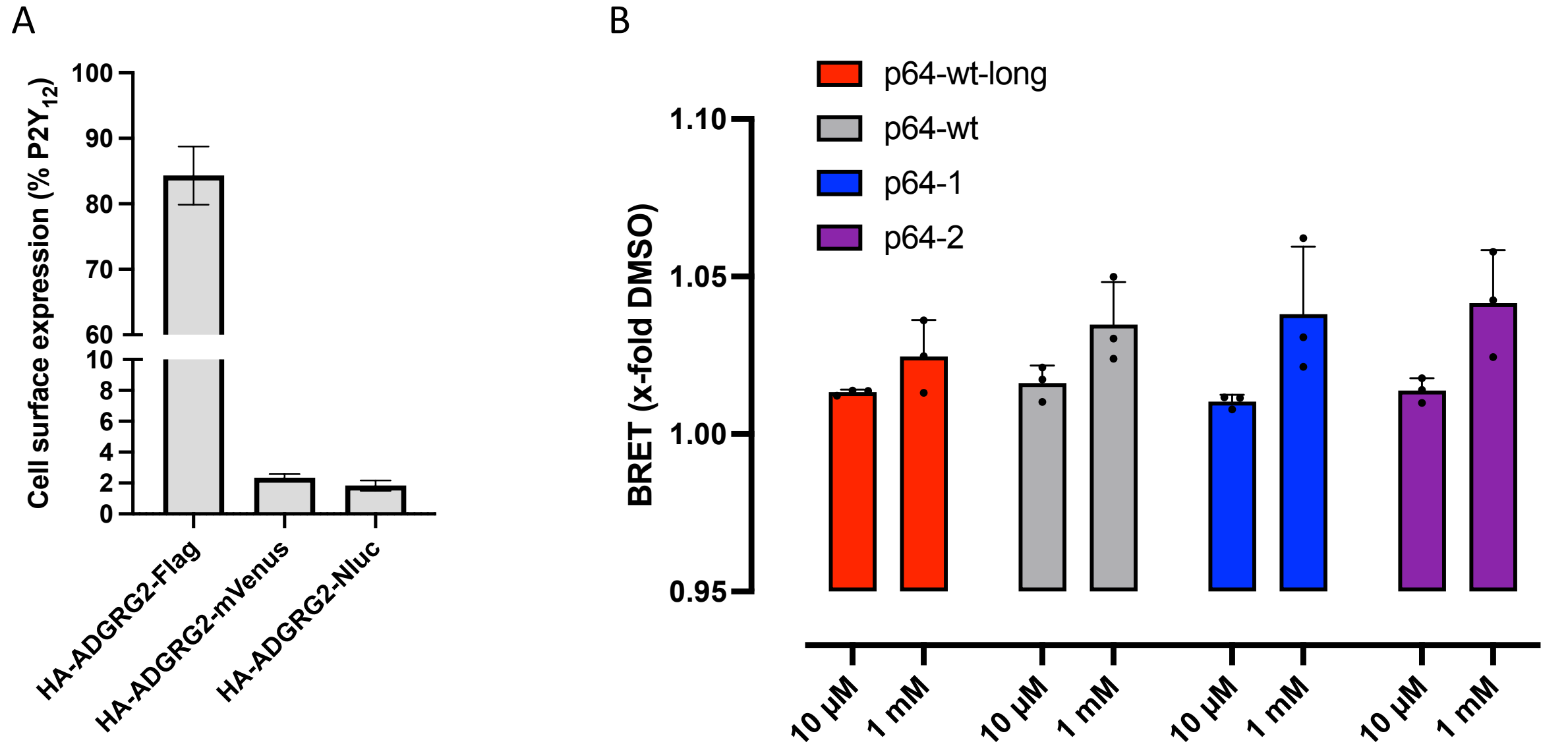

Suppl. Figure S9. Cell surface ELISA and BRET assays for ADGRG2 constructs. (**A**) For the BRET assays, two sets of constructs were generated and tested in HEK293 cells. The mVenus and NLuc reporters were fused to the extreme C terminus of mouse HA-ADGRG2. Cell surface ELISAs were performed using a monoclonal antibody directed against N-terminally HA-tagged ADGRG2, and the data were normalized to N-terminally HA-tagged human ADP receptor P2Y12 (set to 100%). Data are presented as the mean ± S.E.M. from three independent experiments, each performed with technical quadruplicates. (**B**) For the BRET assays, HEK293 cells were cotransfected with an N-terminally HA-tagged mouse ADGRG2-mVenus-N1 construct (ADGRG2-mVenus) and Gs-RLuc at a ratio of 10:1. Two concentrations of the indicated peptides were tested in the BRET assay (5).

Rational design of additional GPR64/ADGRG2-derived agonistic peptides

In the case of ADGRG2-*Stachel*-derived peptides, selection criteria were based on AlphaFold confidence metrics (pLDDT, iPTM and pTM; higher scores indicate better model quality) and on the preferential introduction of polar and charged residues to enhance peptide solubility. Prior knowledge of functionally relevant positions within the peptide was also taken into account when performing manual residue substitutions. The initial objective was to improve the solubility of ADGRG2/GPR64 peptides. For this purpose, the cryo-EM structure 7WUI (mouse ADGRG2) was used as a structural template. A total of 1,000 new sequences were generated, yielding 125 unique peptide sequences. AlphaFold models were generated for all 125 peptides in complex with the 7TM sequence from 7WUI. Models with pLDDT > 80 and iPTM > 0.80 were subjected to visual inspection. The model with the highest pLDDT (92.43) and an iPTM of 0.91 was selected, and the corresponding peptide (p64-3) was chosen for experimental testing (suppl. Fig. S9) after detailed visual structural analysis. To promote hydrogen bonding with residue N666 (numbering according to the 7WUI structure) while preserving hydrophobic packing within the 7TMD binding pocket, position 3 was substituted from phenylalanine (F) to tyrosine (Y), yielding peptide p64-4. With the continued aim of improving peptide solubility, a positively and a negatively charged residue were introduced in two additional variants (p64-5 and p64-6, respectively) (suppl. Fig. S9A); position 10 was chosen for these substitutions because it does not participate in direct interactions at the binding interface. In peptide p64-5, isoleucine was introduced at position 9 together with lysine at position 10; an isoleucine at position 9 was observed in other designs during visual

analysis. An isoleucine was also introduced at position 5 in p64-5 because several designed sequences contained this residue at position 5 and visual inspection suggested that a slightly larger hydrophobic side chain than valine would favor hydrophobic packing within the 7TM pocket.

In a second design strategy, simulations were used to generate 10,000 new sequences based on an AlphaFold model of human ADGRG2 in which the loop covering the 7TM pocket was also modeled. In this round the design objectives were expanded to include not only improved solubility but also enhanced affinity and/or activity. The consensus sequence derived from this approach (peptide p64-1) was selected for experimental testing. Based on prior knowledge and insights from the earlier designs, position 3 in the consensus sequence was again substituted from phenylalanine to tyrosine, yielding peptide p64-2. Finally, to further improve solubility, serine at position 10 was replaced with the negatively charged residue aspartate (D), producing peptide p64-7.

All peptides were tested in ADGRG2-transfected HEK293T cells, and cAMP responses were determined in a concentration-dependent manner (Suppl. Fig. S9B). All newly designed peptides showed good solubility in DMEM or 1% DMSO/DMEM and exhibited agonistic activity. However, no improvement in potency or efficacy was observed compared with the wild-type peptide p64-wt.

A

Ortholog consensus	G2	TSFGVLLDLSR
human	G2	TSFGVLLDLSR
mouse	G2	TSFG**I**LLDLSR
p64-wt-long	G2	TSFGVLLDLSRTSLP
p64-wt	G2	TSFGVLLDLSR
p64-1 (consensus)	G2	**P**SF**T**VLLDLSR
p64-2 (frequent)	G2	**P**S**YT**VLLDLSR
p64-3 (absent)	G2	S**P**F**T**VLLDL**N**R
p64-4 (absent)	G2	S**PYT**VLLDL**N**R
p64-5 (absent)	G2	S**PYT**VLLD**IK**R
p64-6 (absent)	G2	S**PYT**VLLDL**D**R
p64-7 (absent)	G2	**P**S**YT**VLLDL**D**R

B

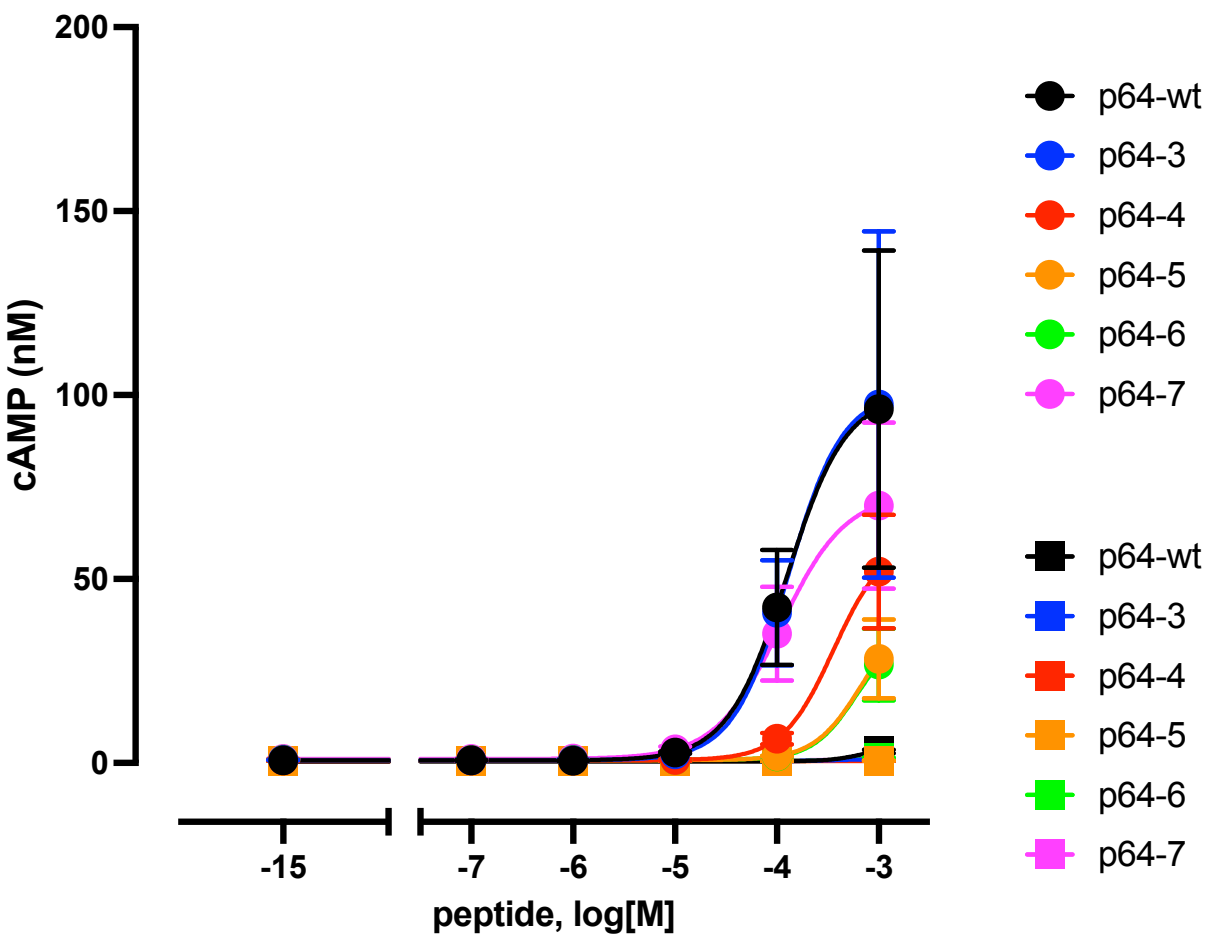

Suppl. Figure S10. Functional analysis of ADGRG2-Stachel sequence variants generated by simulation. (**A**) Alignment of 446 vertebrate orthologs revealed an ortholog consensus sequence identical to the human sequence. Mouse ADGRG2 differs only at position $X^{0.49}$ (V to I), where 81.8% of the investigated orthologs contain valine and 18.2% contain isoleucine. Previous work identified *Stachel* sequence-derived peptides as agonists at the mouse ADGRG2 (4). Based on an available cryo-EM structure of mouse ADGRG2 (PDB: 7WUI), *Stachel* sequence simulations using pMPNN were performed, and peptides were synthesized based on the sequences with favorable *in-silico* scores which were further manually edited to obtain more soluble peptides (see suppl. information). (**B**) HEK293T cells were transiently transfected with mouse ADGRG2, and the indicated peptides were tested in cAMP assays (circles). For control purposes, all peptides were also tested in empty-vector (pcDps)–transfected cells (squares). cAMP levels as a function of peptide concentration are shown. Data are presented as mean ± S.E.M. from two to three independent experiments performed with technical replicates.

A

Ortholog consensus F5 TSFSILMSPDS
human F5 TSFSILMSPDS
mouse F5 TSFSILMSPDS
p116-wt F5 TSFSILMSPDS
p116-1 (consensus) F5 T**PYV**IL**FA**P**EI**
p116-2 (frequent) F5 T**PYV**ILMSP**EI**

B

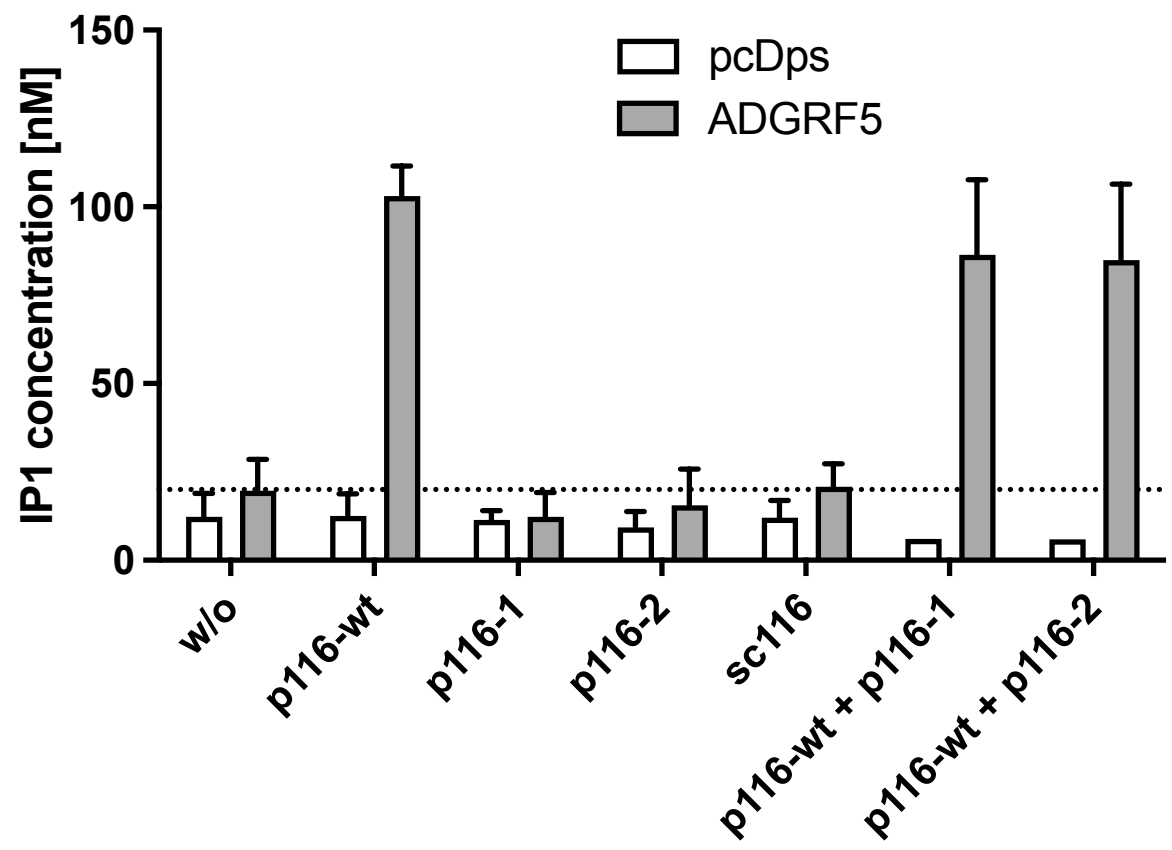

Suppl. Figure S11. Functional analysis of ADGRF5-Stachel sequence variants generated by simulation. (**A**) Alignment of 441 vertebrate orthologs revealed an ortholog consensus sequence identical to the human und mouse sequences. Previous work identified Stachel sequence-derived peptides as agonists at the mouse ADGRF5 (6). Based on an AlphaFold model of the human ADGRF5, *Stachel* sequence simulations using pMPNN were performed, and peptides p116-1 and p116-2 were synthesized based on the consensus sequence and a highly frequent designed sequence. (**B**) HEK293T cells were transiently transfected with mouse ADGRF5 (grey bars), and the indicated peptides (1 mM) were tested in IP1 assays. For control purposes, all peptides were also tested in empty-vector (pcDps)–transfected cells (white bars) and a scrambled peptide (sc116), containing the same amino acid residues as p116-wt but in a different order, was used. Data are presented as mean ± S.E.M. from 3-4 independent experiments performed in technical triplicates.

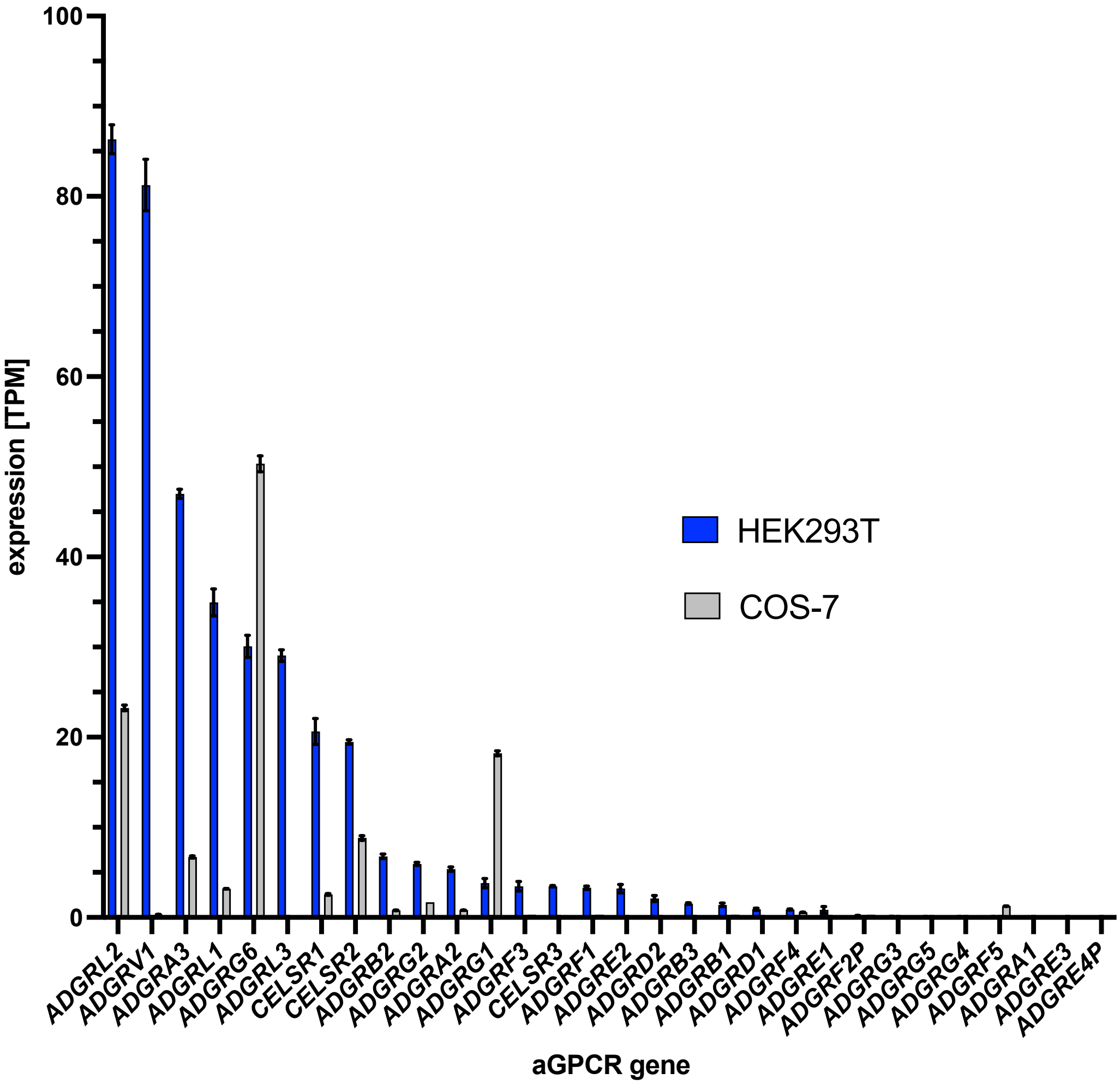

Suppl. Figure S12. aGPCR mRNA quantification in HEK293T and COS-7 cells by RNASeq analyses. Publicly available RNAseq datasets (GSE285962, GSM3431118 (7, 8)) were used to analyze the expression of aGPCRs in HEK293T and COS-7 cells to identify endogenous receptors targeted by p64-2.

Suppl. Table S1. *Amino acid sequences of vertebrate aGPCR used in this study.*

Suppl. Table S2. *Structures used as input models for ProteinMPNN.*

Suppl. Table S3. *Structural variability of* Stachel *sequences in aGPCR ortholog and ProteinMPNN simulations for the GAIN-bound and 7TMD-bound* Stachel*.*

References

1. Scholz, N., Langenhan, T., and Schoneberg, T. (2019) Revisiting the classification of adhesion GPCRs *Ann N Y Acad Sci* **1456**, 80-95
2. Wittlake, A., Promel, S., and Schoneberg, T. (2021) The Evolutionary History of Vertebrate Adhesion GPCRs and Its Implication on Their Classification *Int J Mol Sci* **22**, 11803
3. Kleinau, G., Ali, A. H., Wiechert, F., Szczepek, M., Schmidt, A., Spahn, C. M. T. *et al.* (2023) Intramolecular activity regulation of adhesion GPCRs in light of recent structural and evolutionary information *Pharmacol Res* **197**, 106971
4. Demberg, L. M., Rothemund, S., Schoneberg, T., and Liebscher, I. (2015) Identification of the tethered peptide agonist of the adhesion G protein-coupled receptor GPR64/ADGRG2 *Biochem Biophys Res Commun* **464**, 743-747
5. Liebing, A. D., Rabe, P., Krumbholz, P., Zieschang, C., Bischof, F., Schulz, A. *et al.* (2025) Succinate receptor 1 signaling mutually depends on subcellular localization and cellular metabolism *FEBS J* **292**, 2017-2050
6. Demberg, L. M., Winkler, J., Wilde, C., Simon, K. U., Schon, J., Rothemund, S. *et al.* (2017) Activation of Adhesion G Protein-coupled Receptors: AGONIST SPECIFICITY OF STACHEL SEQUENCE-DERIVED PEPTIDES *J Biol Chem* **292**, 4383-4394
7. Cid-Samper, F., Gelabert-Baldrich, M., Lang, B., Lorenzo-Gotor, N., Ponti, R. D., Severijnen, L. *et al.* (2018) An Integrative Study of Protein-RNA Condensates Identifies Scaffolding RNAs and Reveals Players in Fragile X-Associated Tremor/Ataxia Syndrome *Cell Rep* **25**, 3422-3434 e3427
8. Schindler, J. C., Seth, P., Premont, R. T., and Stamler, J. S. (2025) Transcriptional responses to nitric oxide are widescale and source-dependent *J Biol Chem* **301**, 110476
